# A perspective on the radiopharmaceutical requirements for imaging and therapy of glioblastoma

**DOI:** 10.7150/thno.56639

**Published:** 2021-07-06

**Authors:** Julie Bolcaen, Janke Kleynhans, Shankari Nair, Jeroen Verhoeven, Ingeborg Goethals, Mike Sathekge, Charlot Vandevoorde, Thomas Ebenhan

**Affiliations:** 1 Radiobiology, Radiation Biophysics Division, Nuclear Medicine Department, iThemba LABS, Cape Town, South Africa.; 2 Nuclear Medicine Research Infrastructure NPC, Pretoria, South Africa.; 3 Nuclear Medicine Department, University of Pretoria and Steve Biko Academic Hospital, Pretoria, South Africa.; 4 Laboratory of Radiopharmacy, Ghent University, Ghent, Belgium.; 5 Ghent University Hospital, Department of Nuclear Medicine, Ghent, Belgium.; 6 Nuclear Medicine Department, University of Pretoria, Pretoria, South Africa.

**Keywords:** targeted radionuclide therapy, radiochemistry, glioblastoma, theranostics, PET SPECT imaging

## Abstract

Despite numerous clinical trials and pre-clinical developments, the treatment of glioblastoma (GB) remains a challenge. The current survival rate of GB averages one year, even with an optimal standard of care. However, the future promises efficient patient-tailored treatments, including targeted radionuclide therapy (TRT). Advances in radiopharmaceutical development have unlocked the possibility to assess disease at the molecular level allowing individual diagnosis. This leads to the possibility of choosing a tailored, targeted approach for therapeutic modalities. Therapeutic modalities based on radiopharmaceuticals are an exciting development with great potential to promote a personalised approach to medicine. However, an effective targeted radionuclide therapy (TRT) for the treatment of GB entails caveats and requisites. This review provides an overview of existing nuclear imaging and TRT strategies for GB. A critical discussion of the optimal characteristics for new GB targeting therapeutic radiopharmaceuticals and clinical indications are provided. Considerations for target selection are discussed, i.e. specific presence of the target, expression level and pharmacological access to the target, with particular attention to blood-brain barrier crossing. An overview of the most promising radionuclides is given along with a validation of the relevant radiopharmaceuticals and theranostic agents (based on small molecules, peptides and monoclonal antibodies). Moreover, toxicity issues and safety pharmacology aspects will be presented, both in general and for the brain in particular.

## 1. Introduction

Gliomas represent 80% of all primary brain tumours and are a heterogeneous group of tumours of the central nervous system (CNS). Diagnosis is often predicted by patient clinical history, but confirmation by neuroimaging is required. Before beginning treatment, histological characterisation and determination of the malignancy grade is imperative 1,2. Previously, the classification of CNS tumours by the World Health Organization's (WHO) grading system was solely based on histology; varying from grade I, which is characterised by lesions with low proliferative potential and possibility of cure, up to grade IV. However, several studies over the past two decades illustrate the diagnostic importance of characterising the molecular status of the individual patient's brain tumour. Hence, a new WHO classification, including both, histology and molecular genetic features, was established in 2016 3,4. Glioblastomas (GB) are classified as grade IV CNS tumours; neoplasms which are cytological malignant and mitotically active. They are typically associated with extensive invasion of the surrounding tissue and rapid proliferation commensurate with disease progression 5.

Individuals who are diagnosed with GB have a poor prognosis and the quest for efficient therapy is ongoing. The standard GB treatment consists of debulking surgery, temozolomide (TMZ) chemotherapy and concomitant external beam radiotherapy (EBRT). However, total resection is not possible in most patients. Despite optimal treatment protocols; the median survival is only 12-14 months 6-9. Current therapies fail as the result of therapeutic resistance and heterogeneous tumour cell population effects. GB often presents with different grades of cell-differentiation within the same tumour, indicating the presence of distinct cell populations with differing sensitivity to therapy. Resistance is often caused by the presence of a small subset of highly resistant tumour cells that display stem cell-like properties 10,11.

Target-based diagnostics and therapeutics focus on several mutations and alterations in key molecular pathways that have been linked to GB pathogenesis and/or prognosis. These include, phosphatase and tensin homolog (PTEN) and 1p/19q combined deletions, mutations of the isocitrate dehydrogenase 1 or 2 (IDH) genes and telomerase reverse transcriptase (TERT) promoter region, epidermal growth factor receptor (EGFR) amplification and tumor protein (TP53) mutations 12,13. The advantage of targeting the molecular characteristics that drive the malignant GB phenotype with theranostic radiopharmaceuticals is the possibility of selectively identifying and subsequently treating GB cells without damaging the surrounding healthy brain tissue. The identification of new GB genetic biomarkers has led to a growing interest in the development of new radiopharmaceuticals for GB imaging and therapy 14,15.

TRT is a strategy in nuclear medicine for the treatment of GB enabling the visualization of molecular biomarkers and pathways on a subcellular level using a biochemical vector coupled to a radionuclide either for diagnosis or for therapy. A major prerequisite for the administration of TRT is to confirm the presence of the GB tumour target using non-invasive nuclear imaging techniques before deciding on treatment options. This review includes an overview of current GB imaging options, a detailed perspective on TRT strategies for GB followed by a critical assessment of the TRT requirements to reach optimal treatment outcome in GB patients. Special attention is given to the selection of the optimal target and its accessibility, choice of the biochemical vector, risk for toxicity and desired validation process.

## 2. Nuclear imaging and theranostics in neuro-oncology

Historically, contrast-enhanced magnetic resonance imaging (MRI) played an important role in the diagnosis and the assessment of treatment efficacy in GB. This is still the case, however, the use of contrast enhancement is controversial since it is non-specific and it primarily reflects the passage of contrast material (e.g. gadolinium) across a disrupted blood-brain barrier (BBB). Pseudo-progression is often incorrectly reflected as tumor progression on contrast-enhanced MRI in approximately 20-30% of glioma patients, especially within the first three months after concurrent chemoradiation. In addition, the use of antiangiogenic agents during treatment can result in a 'pseudo-response' on contrast-enhanced MRI 16,17. To accurately assess treatment response, new response criteria for Response Assessment in Neuro-Oncology were introduced in 2010 17. This includes 2D-tumour size as measured on T2- and Fluid Attenuated Inversion Recovery (FLAIR)-weighted MR images, in addition to contrast-enhanced MRI.

Non-invasive, functional and molecular imaging techniques have become recognised as more relevant in the last decade, including MR spectroscopy, perfusion weighted MRI, Positron Emission Tomography/Computed Tomography (PET/CT) or Single-photon Emission Computerized Tomography (SPECT/CT). PET has a clear advantage over SPECT in terms of spatial resolution and is therefore the image modality of choice regarding GB investigations. Imaging gliomas using PET has been reviewed in depth elsewhere 18-21. When a theranostic approach is used for GB treatment, the major role of PET or SPECT includes confirmation of the presence of the specific molecular target before TRT. Carefully interpreted Nuclear Medicine imaging facilitates the prediction and monitoring of tumour response and individualised dosimetry 22-24. Biodistribution analysis of the imaging partner permits improved patient-based treatment and thereby prevents unnecessary therapy and associated toxicity 25. This may be achieved by, for example, exchanging the therapeutic radionuclide (e.g. β-emitter Lutetium-177) with a gamma- or positron-emitting radionuclide (e.g. Gallium-68 for PET/CT) attached to the relevant biomolecule; ie. using [^68^Ga]Ga-DOTA-TOC-PET/CT imaging combined with [^177^Lu]Lu-DOTA-TATE-targeted radionuclide therapy 26. Another approach is to use a solitary radionuclide that emits both therapeutic and imageable γ-rays or positrons that allows GB imaging using SPECT or PET (e.g. Iodine-131) 7.

### 2.1 Established and emerging PET/SPECT radiopharmaceuticals in neuro-oncology

**Figure 1:**
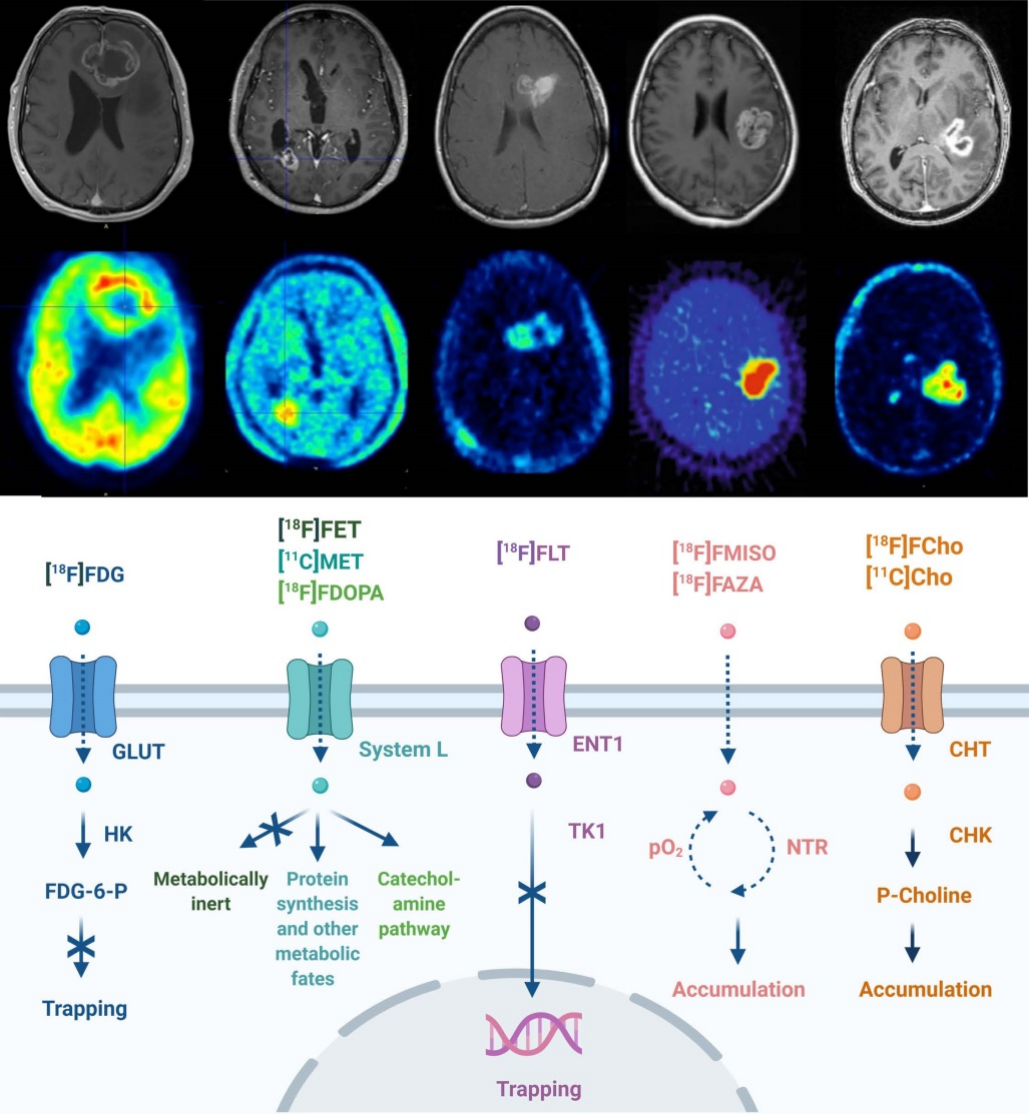
** Routine PET imaging in neuro-oncology.** PET/CT techniques for neuropathologic imaging are dominated by radiopharmaceuticals focussing on altered glucose metabolism (desoxy-2-[^18^F]fluoro-D-glucose ([^18^F]FDG)), amino acid metabolism (L-[^11^C]-methyl-methionine ([^11^C]MET), O-2-[^18^F]fluoroethyl-L-tyrosine ([^18^F]FET), 3,4-dihydroxy-6-[^18^F]fluoro-L-phenylalanine ([^18^F]F-DOPA)), proliferation (3'-deoxy-3'-[^18^F]fluoro-thymidine [^18^F]FLT)), tumoral hypoxia sensing ([^18^F]fluoro-misonidazole ([^18^F]FMISO), [^18^F]fluoro-azomycin arabinoside ([^18^F]FAZA)), and lipid metabolism ([^11^C]choline ([^11^C]Cho), [^18^F]fluoroethyl-choline ([^18^F]FCho)). Abbreviations: High affinity choline transporter (CHT), choline kinase (CHK), equilibrative nucleoside transporter (ENT), 2'-fluoro-2'-deoxy glucose-6-phosphate (FDG-6-P), glucose transporter (GLUT), hexokinase (HK), nitroreductase (NTR), partial pressure of oxygen (pO_2_), Na+-independent plasma membrane amino acid transport (System L), thymidine kinase (TK). Adapted with permission from 18, copyright 2017 Codon Publications.

The transport and cellular mechanism of routinely used PET tracers in neuro-oncology is given in **Figure 1.** GB PET radiotracers are predominantly biomimetics excessively incorporated by cancer cells in response to elevated metabolism or high proliferation. These may include desoxy-2-[^18^F]fluoro-D-glucose ([^18^F]FDG), L-[^11^C]methyl-methionine ([^11^C]MET), O-2-[^18^F]fluoroethyl-L-tyrosine ([^18^F]FET), 3,4-dihydroxy-6-[^18^F]fluoro-L-phenylalanine ([^18^F]FDOPA) and 3'-deoxy-3'-[^18^F]fluoro-thymidine ([^18^F]FLT) 20,27-29. Whilst [^18^F]FDG PET is widely available, the high physiological brain uptake of glucose and the non-specific uptake in cerebral inflammatory processes hampers applications of [^18^F]FDG PET for brain tumor delineation and diagnosis. Amino acid radiopharmaceuticals designated for PET have improved diagnostic glioma PET imaging towards the delineation of tumor extent, treatment planning, visualization of treatment-related changes and the assessment of treatment response 21. PET radiopharmaceutical choline analogues are considered successful as oncological PET probes because a major hallmark of cancer cells is increased lipogenesis. In the brain, discrimination between tumor and normal tissue is feasible because of lower physiological uptake of [^11^C]choline ([^11^C]Cho) or [^18^F]fluoroethyl-choline ([^18^F]FCho) by normal brain cells 30,31. The performance of [^18^F]FCho-PET may distinguish high-grade glioma, brain metastases and benign lesions in addition to its importance for surgery management (including identifying the most malignant areas for stereotactic sampling) 32-35. As hypoxia plays an important role in GB pathology, its detection and monitoring using PET/SPECT became clinically relevant. Radiopharmaceuticals used for these investigations include ([^18^F]fluoro-misonidazole ([^18^F]FMISO), [^18^F]fluoro-azomycin arabinoside ([^18^F]FAZA), [^18^F]fluoro-erythro-nitroimidazole ([^18^F]FET-NIM), 2-(2-nitro-1-H-imidazol-1-yl)-N-(2,2,3,3,3-penta-[^18^F]fluoropropyl)-acetamide ([^18^F]EF5), [^18^F]flortanidazole ([^18^F]F-HX4), and Copper(II)-[^64^Cu]diacetyl-di(N4-methylthiosemicarbazone ([^64^Cu]Cu-ATSM)) 36.

An exhaustive list of the emerging PET and SPECT radiopharmaceuticals, matched with their biological targets, is summarized in **Table 1**. The rational for the use of selected examples is described briefly as follows: The translocator protein (TSPO) is a mitochondrial membrane protein highly expressed in activated microglia, macrophages, and neoplastic cells. Imaging with the TSPO ligand [^11^C]-(R)PK11195 demonstrated increased binding in high-grade glioma compared to low-grade gliomas and normal brain parenchyma in patients 37. [^18^F]F-GE-180-PET further provided a remarkably high tumour-to-background contrast in GB 38. Radiolabeling of poly ADP ribose polymerase (PARP) inhibitors is gaining interest with numerous preclinical studies and an ongoing clinical trial in GB patients using [^18^F]-FluorThanatrace ([^18^F]F-TT)-PET/CT (NCT04221061) 36,39-43. The first clinical results of [^18^F]-Fluciclovine ([^18^F]F-ACBC) for GB imaging were promising and radiolabelling of receptor tyrosine kinase inhibitors and mammalian target of rapamycin (mTOR) pathway inhibitors has also shown potential 44-51. It is noted that PET imaging using the deoxycytidine kinase substrate [^18^F]F-clofarabin has been shown to be a good imaging tool to localise and quantify responses in GB patients undergoing immunotherapy 52. In addition to their application as diagnostic biomarkers, the use of theranostic (pairs of) radiopharmaceuticals that enable concomitant or subsequent imaging and therapy is gaining importance although not all have been validated in clinical trials. The αvβ3 integrin receptor-targeting agent AI[^18^F]F-NOTA-PRGD2 showed positive results in assessing sensitivity to concurrent chemoradiotherapy in GB patients. Therapeutic radionuclides coupled to arginine-glycine-aspartate (RGD) based vectors, already available, offer potential theranostic applications to target tumour angiogenesis 53. The theranostic potential of [^64^Cu/^67^Cu]Cu-cyclam-RAFT-c(RGDfK)_4_ to treat GB *in vivo* shows promise 54-56. Moreover, as the Food and Drug Administration (FDA) approved somatostatin receptor 2 (SSR2) targeting, gallium-68-labeled octreotide derivatives were approved ([^68^Ga]Ga-DOTA-TOC; alternately [^68^Ga]Ga-DOTA-NOC and -TATE are utilized) and subsequent studied for GB imaging. However, their specificity and selectivity towards GB have not yet been clinically determined 57,58. Nevertheless, pilot studies in glioma patients with gallium-68- and yttrium-90-labeled SSTR2-targeting ligands, have been performed 59-62. Additionally, a fibroblast activation protein inhibitor (FAPI) labelled with gallium-68 ([^68^Ga]Ga-FAPI) was introduced into clinical investigations and exhibited significant uptake in IDH-wildtype GB tumours, grade III and grade IV IDH-mutant gliomas. FAPI-targeted theranostics (pairing or gallium-68 and yttrium-90 or gallium-68 and lutetium-177) were developed. However, due the short retention time, radionuclides with shorter half-lives (e.g. rhenium-188, samarium-153, bismuth-213 or lead-212) appeared preferable 63-65. Furthermore, a growing number of copper-based PET tracers are being studied for use in GB investigations, with the emerging theranostic copper-64 and copper-67, characterised by a joint positron/auger electron and joint beta/gamma emission, respectively. In patients, PET imaging using [^64^Cu]CuCl_2_ has visualized brain cancerous lesions and initial investigations using [^64^Cu]Cu- or [^62^Cu]Cu-ATSM-PET imaging may address the hypoxia status of GB, non-invasively 66-69. Preclinically, ^64^Cu-labelled peptides and ^64^Cu-labeled cetuximab have shown promise in imaging of VEGFR and EGFR expression, respectively 70-74. Other preliminary theranostic applications studied *in vivo* include [^64^Cu]Cu-ATSM, [^64^Cu/^67^Cu]Cu-cyclam-RAFT-c(RGDfK)_4_ (αVβ3 integrin), [^64^Cu]Cu-PEP-1L (IL-13 receptor) and [^64^Cu]Cu-IIIA4 (ephrin type-A receptor 3) 55,56,70-77. Interestingly, prostate-specific membrane antigen (PSMA) expression has been confirmed in the neovasculature of GB and the diagnostic role of radiolabelled PSMA PET/CT or PET/MRI in patients with gliomas and GBs has recently been reviewed 78-81. In particular, the radiolabeled ligand [^68^Ga]Ga-Glu-urea-Lys(Ahx)-HBED-CC ([^68^Ga]Ga-PSMA-11) has shown positive results in visualizing residual or recurring GB 82,83. A proof of concept for the theranostic potential of [^68^Ga]Ga-PSMA-11/[^177^Lu]Lu-PSMA-617 in GB has demonstrated success in 2 recent case reports 84,86. However, large prospective studies are needed to clarify the diagnostic role of the radiolabeled PSMA ligands in GB imaging. To date, some studies are featuring imaging of cerebral cancer using novel [^89^Zr]Zr-/[^18^F]F-labelled PSMA compounds; however, the preclinical applications particularly using GB animal models are limited to one study 87-91.

### 2.2 Selection of the appropriate theranostic pair for individualised treatment

Diverse information summarised in **Table 1**, demonstrates that a broad spectrum of investigations in the field of neuro-oncology imaging are well underway. Despite the development of a variety of imaging strategies, evident in **Table 1** for example, only the most effective will be evaluated in clinical trials and, if deemed appropriate, become routinely available in Nuclear Medicine. Theranostics and nanotheranostics which include the future of theranostics and precision oncology are reviewed 22,94,95; for such endotherapies, visualization of GB tumour tissue is critical to predict prognosis accurately including loss in brain function. Additionally, the tracer coupled to the therapeutic radionuclide and the imaging radionuclide should not alter the drug's binding, pharmacokinetics or BBB crossing characteristics. **Table 2** lists targeted radionuclides and theranostic pairs appropriate for GB, including their advantages and disadvantages. Examples for GB include [^68^Ga]Ga-DOTA-SP co-injection with [^213^Bi]Bi-DOTA-SP to assess the biodistribution using PET/CT and [^68^Ga]-pentixafor-PET/CT as a tool for *in vivo* quantification of CXCR4. This will facilitate the selection of patients who might benefit from CXCR4-directed therapy. Another example is [^131^I]-labeled anti-tenascin murine 81C6 mAb SPECT to assess the distribution of the radiolabeled mAb in brain parenchyma 93-96.

## 3. Selection of the optimal target for imaging and TRT of GB

An important consideration for the selection of a GB target for TRT is the abundance of the molecular target present in the tumour versus its negligible presence in normal cells. The target must have proven of relevance for therapy and the finally selected compound must demonstrate bioequivalence at the target site and the radiopharmaceutical must be retained within the tumour. The pathology of most GB tumours is not based on the dysregulation of a single pathway and therefore, a strategy with a multi-targeted design should also be considered 97. For a more detailed explanation of the principles of optimal target selection for diagnostic, therapeutic and theranostic applications in nuclear medicine, please refer to the review by Lee *et al*. 98.

### 3.1 GB target abundance, stability and specificity

Large-scale genomic (Cancer Genome Atlas (TCGA)) and proteomic analysis of GB tumours have uncovered potential targets that are deemed relevant to both imaging and therapy 99,100. Abundantly expressed targets reduce the absolute need for a radiopharmaceutical to have high molar activity (MA). Furthermore, due to the correlation between specific activity and MA, this provides the opportunity to use radionuclides with lower specific activity for the radiosynthesis. Antigenic targets are usually tumour cell surface-expressed macromolecules, which are easily accessible by compounds present in the blood pool or extracellular fluid. In the case of GB, this includes cell surface glycoproteins 101,102, enzymes such as PSMA 79,80, glycolipids 103, stromal components 11, components of blood vessels (e.g. VEGF) 104 and signal transduction molecules (e.g. growth factor receptors) 97. As an example, the target of substance-P (SP), the neurokinin-1 receptor, is an appropriate target due to the high prevalence on the membrane of GB cells with strong expression on the tumour vasculature, allowing concomitant dual targeting 105. Another example is tenascin, an extracellular matrix glycoprotein overexpressed by GB and minimally presented in normal tissue with a significant role in angiogenesis, which demonstrated encouraging results in TRT trials with GB 106. As the tumour microenvironment (TME), hypoxia and glioma stem cells are pivotal in GB progression and resistance, their cell surface markers and specific pathways present attractive and important target options 5,10,11,107.

A homogeneous antigen expression and a very high affinity of the drug to the target are more important for α-emitting and AE-emitting radiopharmaceuticals, due to the fact that there is no cross-fire effect. For radio-immunotherapy (RIT), the antigen expression should be >100000 sites per cancer cell with a uniform density on the surface of all tumour cells, no expression on normal cells, and no dispersion into the bloodstream 108. The choice of the vector is another challenge as antibodies provide the highest total in-tumour accumulation, while smaller molecules such as peptides provide the highest tumour-to-normal organ dose ratios 109,110.

Another desirable aspect for successful imaging and effectiveness of TRT is the degree of receptor internalisation (or of other surface macromolecules) upon binding, causing continued accumulation of the radionuclide intracellularly. Phenotypic instability is a reason for caution as complex epigenetic factors exist which can upregulate or downregulate target activation. The theranostic approach is particularly useful since it allows the visualisation and quantification of the specific molecular target during planning of the adequate therapeutic approach and more importantly during therapy follow-up. The necessity for continuous validation of target expression in GB therapy is considered in the ACT IV trial on rindopepimut in patients with EGFRvIII-positive GB. This study showed a striking loss of EGFRvIII expression at recurrence in both groups of the trial, suggesting that EGFRvIII expression is unstable, which could limit its use as a target for TRT 97.

**Figure 2:**
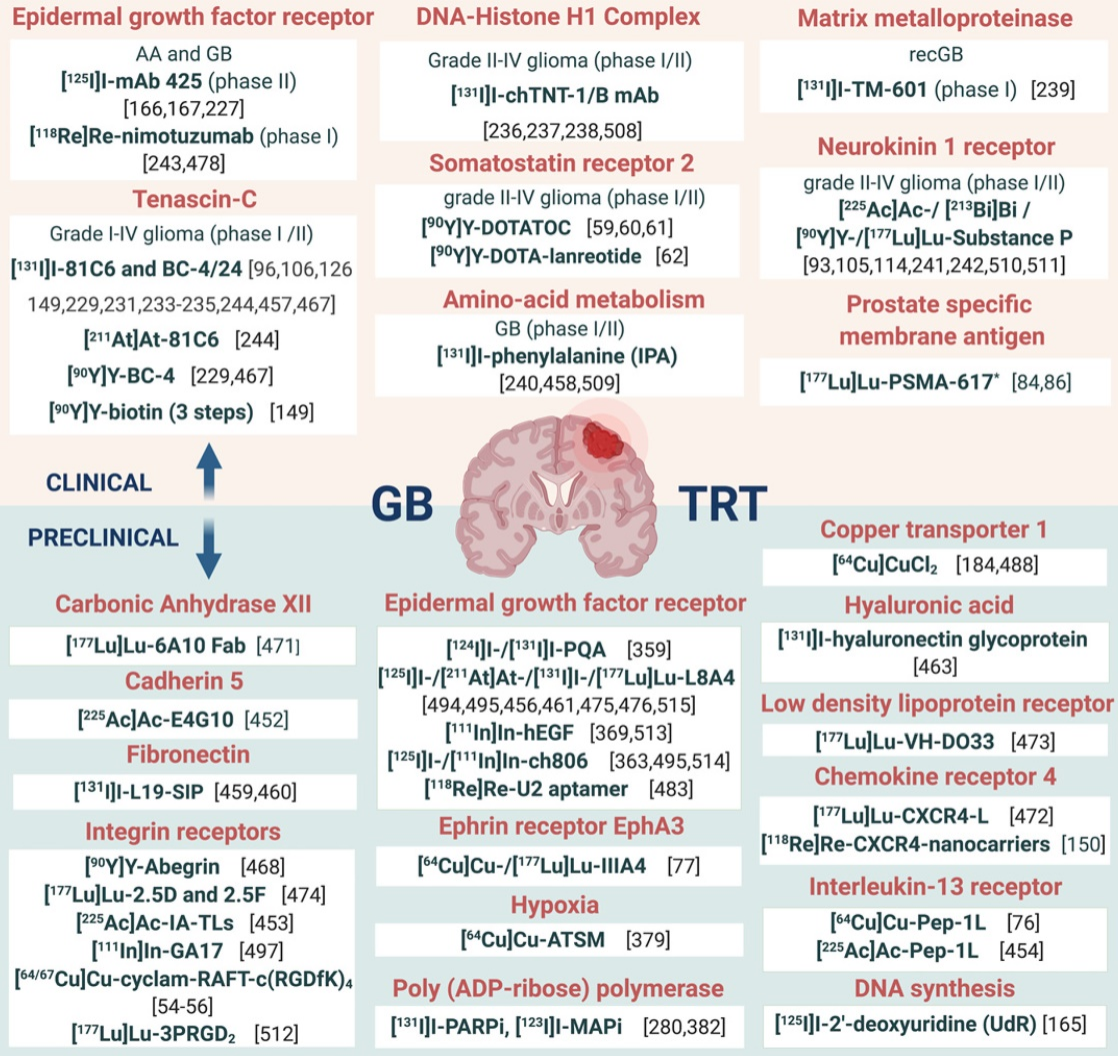
** Overview of current clinical and preclinical targeted radionuclide therapy studies in glioblastoma.** Abbreviations and footnoted content: Anaplastic astrocytoma (AA), convection enhanced delivery (CED), glioblastoma (GB), recurrent (rec), deoxyribonucleidic acid (DNA), monoclonal antibody (mAb), targeted radionuclide therapy (TRT), (*) human case study 46,54,55,59-62,76,77,84,86,93,96,105,106,114,126,149,150,165-167,184,227,229,231,233-244,280,359,363,369,379,382,452-454,456-461,463,467,468,471,472,473,476,478,483,488,494,495,497,508-515.

In addition, it should be noted that the interplay of receptors, compound binding and cellular uptake pathways may cause receptor saturation upon injection of therapeutic doses. TRT approaches for GB treatment are gaining momentum and have been reviewed 14,111,112. However, in this review, an extensive overview of prospective targets on GB is presented (**Figure 2**). Other indications for TRT in neuro-oncology, include grade I-III glioma (e.g. radiolabeled SP and anti-EGFR TRT), brain metastasis (e.g. ^177^Lu]Lu-/[^225^Ac]Ac-PSMA-617), meningioma (e.g. radiolabelled DOTA-TOC and DOTA-TATE), lymphoma (e.g. [^90^Y]-ibritumomab tiuxetan) and neuroblastoma (e.g. [^131^I]iodo-MIBG) 113,114.

When the identified targets in GB are compared with recent reviews listing current targeted therapies for GB, many possible strategies, which have received little attention, exist for imaging and TRT of GB 7,15,97,115. Such unexplored pathways include: the phosphatidylinositol 3-kinase/Akt/mTOR pathway, the cell cycle pathway, the DNA repair pathway, the notch pathway, the hypoxia pathway and immune checkpoints. Unfortunately, issues such as specificity, selectivity, sensitivity, and feasible radiochemistry (especially molecular stability) challenge the design and synthesis of radiopharmaceuticals 116.

### 3.2 Blood-brain barrier permeability

**Figure 3:**
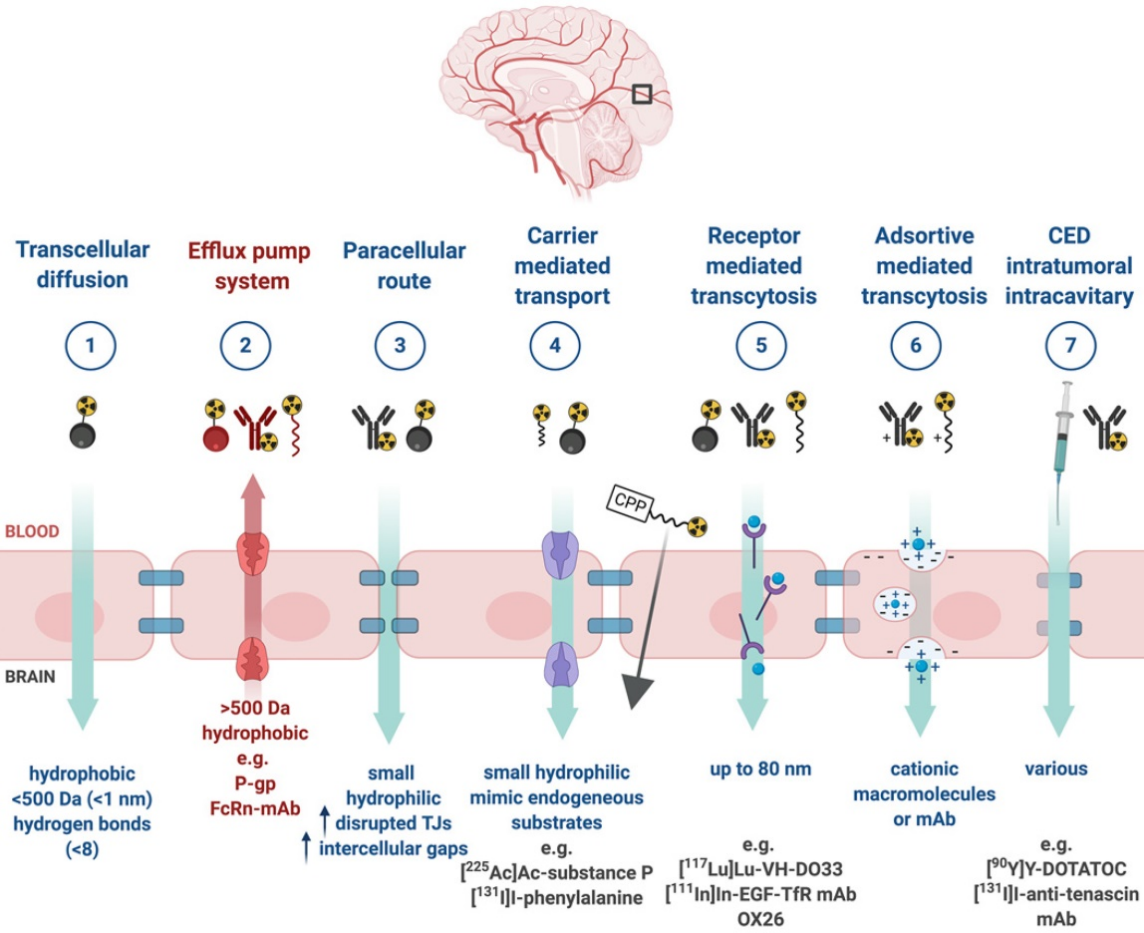
**Mechanisms for transport of radiopharmaceuticals across the blood-brain barrier.** Abbreviations: convection enhanced delivery (CED), cell-penetrating peptides (CPP), monoclonal antibody (mAb), P-glycoprotein (P-gp), tight-junction (TJ).

Upon successful target selection, the most important factor in managing GB is the ability of the designed radiopharmaceutical to cross the BBB and reach this target. Failure to adequately circumvent the BBB and heterogeneous perfusion to the tumour could be partially responsible for any suboptimal compound delivery to brain tumours and the lack of tangible progress in the implementation of targeted therapeutics 117. **Figure 3** gives an overview of the different mechanisms to cross the BBB, including passive mechanisms (1-3), mediated mechanisms (4-6) and a strategy to bypass the BBB (7) 118,119. The mechanism is significantly affected by the choice of the vector, i.e. radiolabelled small molecules, peptides or monoclonal antibodies (mAbs). Small molecules have multiple options to cross the BBB while antibodies are very limited (0.1-0.2%/ID) 12,92,119-123. Compromised integrity of the BBB is a pathophysiological component of GB infiltration which influences the passage of radiopharmaceutical drugs, by increasing the fraction of paracellular diffusion (**Figure 3** (**3**)). Importantly, this increased BBB permeability is dynamic, heterogeneous and can be absent along the infiltrating edges of the GB tumour 5,117,118. This is confirmed by contrast-enhanced MRI where often not all GB components are characterized by gadolinium uptake, which represents leakage. Affinity for efflux transporters can counteract the uptake across the BBB (**Figure 3** (**2**)) and it should be noted that compound assortment by any existing intact BBB transport will be performed regarding enantiomers of several PET-radiopharmaceuticals (small molecules) 118,123. Hence, the radiopharmaceutical design needs to be well adjusted and may have to account for an enhanced BBB passage 124. Even when the radiopharmaceutical is capable of crossing the BBB, diffusion and distribution throughout the GB tumor will be encumbered by an increased interstitial pressure, pooling in excessive (central) necrotic tissue or cystic regions, or by close proximity to ventricles 105.

### 3.3 Strategies to enhance general pharmacokinetics and BBB penetration

**Figure 4:**
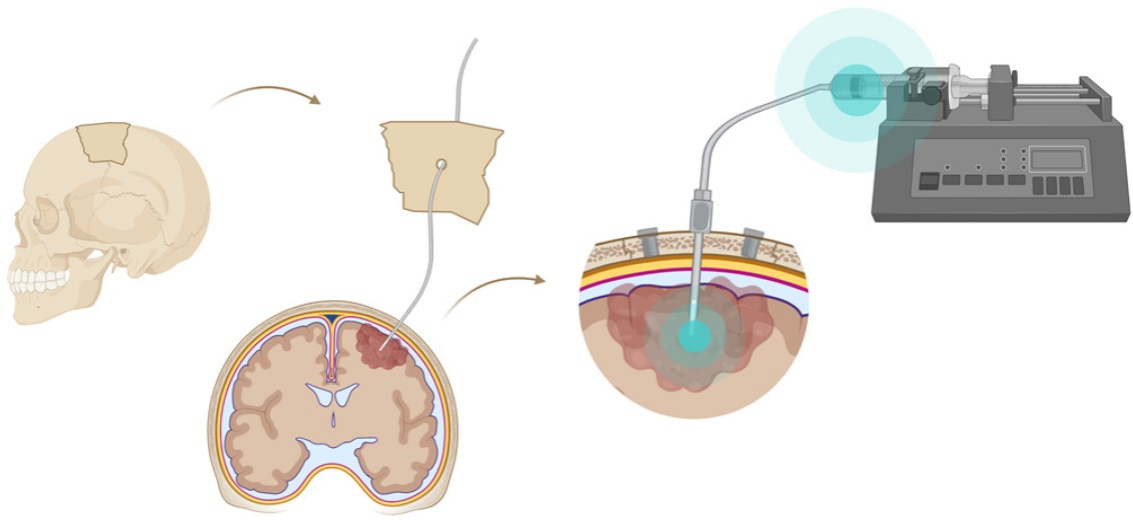
**Convection enhanced delivery (CED) of a radiopharmaceutical.** CED is a strategy whereby a drug is delivered directly into the tumor parenchyma via implanted catheters. Catheters are coupled with a pump to provide continuous positive-pressure microinfusion. Unlike systemic therapy, CED bypasses the blood-brain barrier (BBB) therefore making drug distribution relatively independent of its molecular charge and size 129.

**Figure 5:**
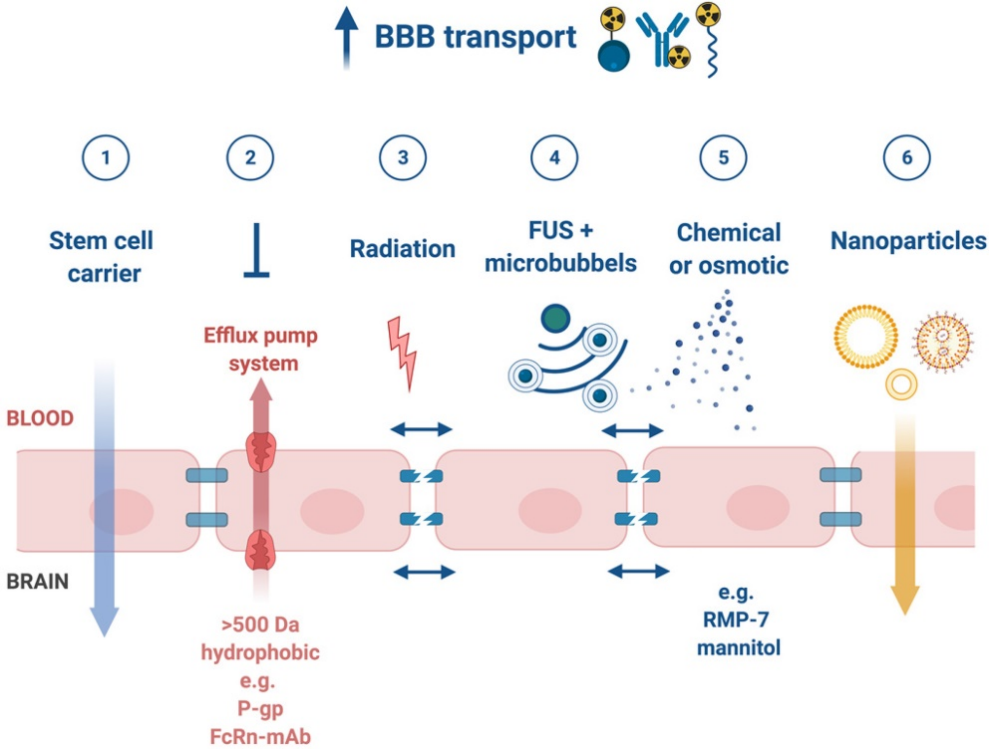
** Strategies to enhance blood-brain barrier (BBB) penetration.** (1) harnessing the homing ability of certain stem cells, (2) low affinity to efflux pumps or co-administration with inhibitors of efflux pumps, (3) targeted irradiation, (4) a combination of low-intensity focused ultrasound (FUS) pulses and circulating microbubbles, (5) infusion of hypertonic solutions, such as mannitol or vasodilatator and bradykinin analog RMP-7 and (6) nanoparticle-mediated delivery systems 118.

A very successful strategy to bypass the BBB for GB TRT is loco-regional compound injection or convection enhanced delivery (CED). This is possible because 95% of GBs manifest as a unifocal lesion that recurs within a 2 cm margin at the primary site 105. Most clinical RIT studies for malignant gliomas were performed via local administration 104,125,126. Human studies using locoregional administration also showed promise in terms of tumour cell incorporation of AE-emitters 127. In a clinical study by Krolicki *et al*., local injection of [^213^Bi]Bi-DOTA-SP was successfully performed 2-4 weeks after stereotactic implantation 105. This group recommends an injection of corticosteroids and antiepileptic drugs thirty minutes before administration and up to 3 mL of injection volume. Co-injection of an imaging and therapeutic radionuclide (e.g. [^68^Ga]Ga-DOTA-SP combined with either [^213^Bi]Bi-DOTA-SP or [^225^Ac]Ac-DOTA-SP) enabled its distribution in the tumour to be monitored and subsequently the radioactivity occurrence in the whole body 105. For CED, a catheter system, stereotactically placed intratumourally or into the post-surgical cavity, employs a pump to provide continuous positive pressure for local drug delivery (ranging from 0.1 to 10 μl/min) (**Figure 3** and **Figure 4**) instead of a bolus injection 128,129. This was proved to be a safe and effective drug delivery method, reaching a higher concentration of the drug within the GB tumour, and lack of systemic toxicity. This is especially favourable for α-particle emitters with relatively short half-lifes, such as bismuth-213 (45 min) or astatine-211 (7.2 h), as most of the radioactive decay will occur within the relevant cavity before being distributed throughout the body via the systemic and lymphatic systems 130. Clinical trials applying CED are highlighted in recent reviews 111,131,132. It should be noted that pre-therapy PET or SPECT imaging following traditional IV tracer injection contributes little information regarding TRT agent distribution, if CED is applied. When the position of a critical lesion makes local application of CED impossible, brain delivery of radiopharmaceuticals can still be improved by different strategies. In addition to the transcellular lipophilic pathway, the use of BBB shuttles constitute an elegant strategy to target the brain, including receptor-mediated transcytosis (RMT), carrier-mediated transcytosis (CMT) or adsorptive-mediated transcytosis (AMT) (**Figure 3**) 118,133. RMT is another elegant strategy for the delivery of macromolecular pharmaceuticals (up to 80 nm in diameter) in the treatment of GB. However, the widespread expression of these receptors in other tissues, the small dissociation rate and potential toxicity require careful consideration 5,118. Alternatively, relevant strategies modifying the PK of radiopharmaceuticals were recently reported 134,135. Chimeric cell-penetrating peptides (CPP) can hereby aid the transportation of drugs (also tumour targeting peptides) unable to pass the BBB, by conjugating it to a brain drug-targeting vector. This CPP complex can cross the BBB via transcytosis; Mendes *et al*. reviewed this aspect for applications for GB therapy (**Figure 3**) 5. Multiple prodrug strategies have been employed to facilitate transport into the CNS for brain tumour visualization and treatment, for instance carrier/ligand-drug conjugates 102. The brain drug-targeting vector can be an endogenous peptide, a modified protein, or a peptidomimetic mAb that undergoes RMT through the BBB on endogenous receptor systems. One such example is [^111^In]In-EGF-SPECT-imaging, using a radiolabeled peptide conjugated to the transferrin receptor (TfR) targeting mAb OX26, which has been shown to detect brain tumours without EGF transport 136. The diagram in **Figure 5** demonstrates other strategies to increase BBB penetration. A fractionated dose administration over time could be advantageous to accommodate changes in blood flow and reductions in interstitial pressure caused by tumour reduction (**Figure 5 (3)**) 137. A physical approach is the combination of low-intensity focused ultrasound (FUS) pulses with circulating microbubbles, which enhanced brain tumor delivery of trastuzumab, improving survival in a rat glioma model (**Figure 5 (4)**) 118,133,138. The issue of a limited BBB penetration of mAbs, due their molecular size and hydrophilicity, may be overcome by using smaller antibody fragments or engineered antibodies 139. Other noteworthy delivery platforms shuttling antibodies to the brain (tumour) may include liposomes, nanoparticle-based systems, CPPs, and whole cell-based concepts, actively studied in GB 5,140-146. This can be combined with a pre-targeting approach, i.e. the administration of a non-radiolabeled antibody first, allowing it to localise to solid tumour sites, followed by a subsequent administration of a small molecular weight, radioactive moiety with high affinity for the tumour reactive antibody 108. This strategy was successful using a three step ytterium-90 labelled biotin-anti-tenascin-PRIT approach in glioma. However the significant immunogenicity of streptavidin may cause negative side effects 147-149. An active targeting approach, such as the encapsulation in polymeric nanocarriers, can be used to optimise confinement of the radioactivity near the GB cells (including daughter atoms) (**Figure 5 (6)**) 5,14,150. The latter resulted in positive pre-clinical results 150,151. In clinical studies of high-grade gliomas (treated with liposomal doxorubicin plus RT and TMZ) limited therapeutic efficacy was evident 118,150-152. Finally, it should be noted that translation of nanoparticle-mediated delivery systems to the clinic is time-consuming, costly, and difficult.

## 4. Requirements for a successful radionuclide therapy agent in glioblastoma

In the last decade, TRT has shown not only to be useful in a palliative context but also to prolong progression-free, overall survival and improve the quality of life of cancer patients 22. Despite a general success of TRT implementation for numerous human investigations, such as GB therapy, and some unparalleled treatment responses, universally applicable guidelines and requirements addressing the use of such theranostic radiopharmaceuticals are yet to be established.

### 4.1 Selection of the radionuclide, optimal LET and range

**Figure 6:**
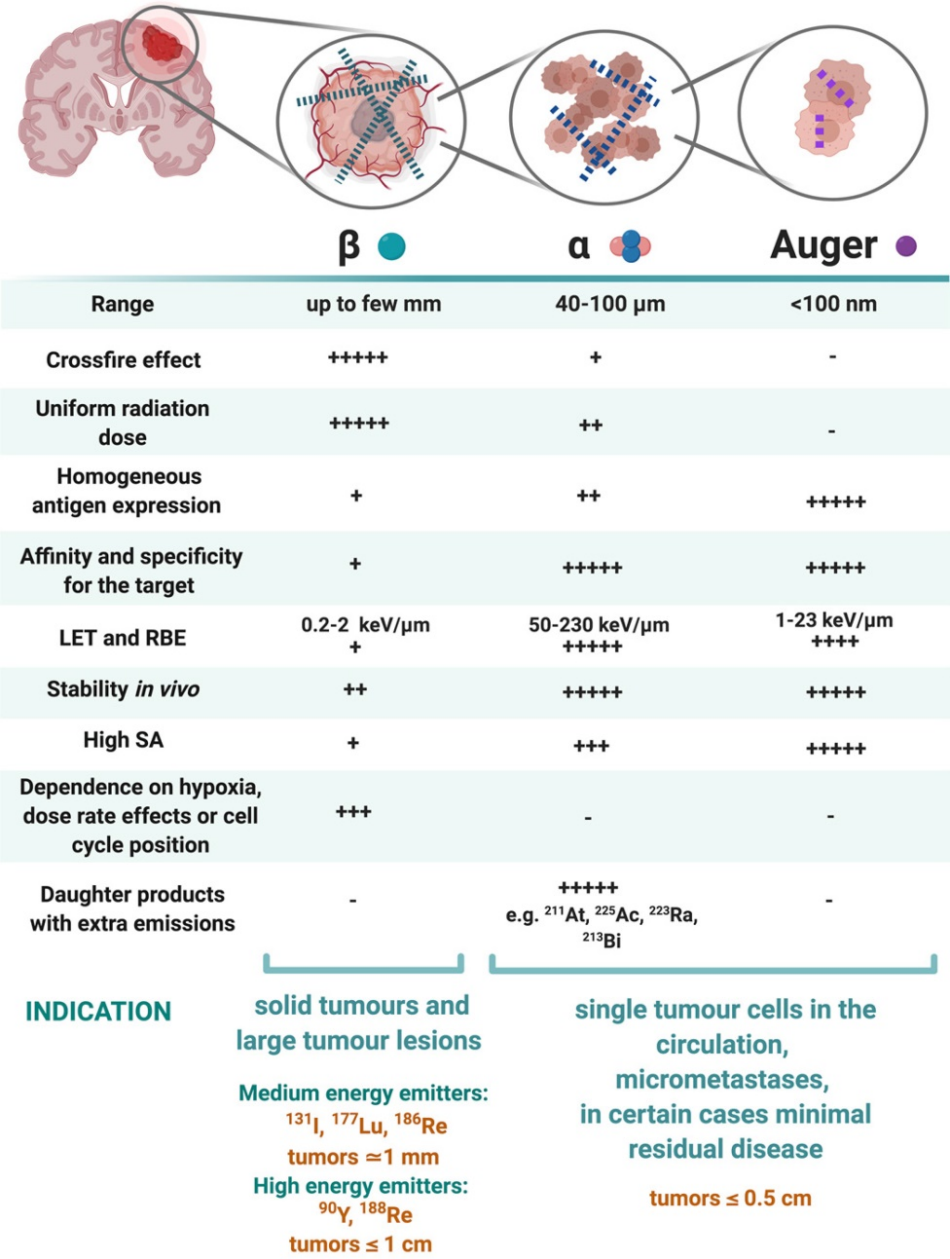
**Characteristics of β-emitting radionuclides versus α particle- and Auger electron-emitting radionuclides.** Abbreviations: Linear energy transfer (LET), relative biological effectiveness (RBE), specific activity (SA) 130,147,155,156.

The three types of radionuclides considered for TRT of GB are α-, β- and Auger electron-emitting radionuclides. Lutetium-177, iodine-131, rhenium-186, rhenium-188 or yttrium-90, are commonly utilized for the treatment of GB. However, targeted α-particle therapy (TAT) using astatine-211, actinium-225 or bismuth-213, is gaining momentum 153. The physical properties and the advantages versus disadvantages of relevant therapeutic radionuclides, in particular for GB TRT, are summarised in **Table 2** and** Figure 6.** Matching the radionuclide correctly (including decay pathway, effective tissue range, linear energy transfer (LET) and relative biological effectiveness (RBE)) to tumour characteristics (size, radiosensitivity and level of heterogeneity) is one of the primary considerations to maximise therapeutic efficacy in TRT 153-155. The extent and location of the GB tumour in the pre-therapy state or after surgical debulking is another major factor influencing the selection process of the appropriate radionuclide (type and/or energy), hence the importance of PET/SPECT imaging to investigate the state of therapy.

*β-emitting radionuclides,* such as iodine-131 and yttrium-90, are used in approximately 90% of current clinical TRT applications 154. Their cross-fire effect (100-300 cell diameters) and relatively long range (0.2-12 mm) make them particularly efficient for the treatment of common bulky, heterogeneous primary (not necessitating homogenous distribution) and recurrent GB with an average size of >0.5 cm. The variety of β-emission ranges with different energies promotes tailoring of treatment to the size of the brain tumour (**Table 2**) 154-156. For example, yttrium-90 (max range 12 mm) could be used for medium-large GB masses, while lutetium-177 (range 2 mm) would be a favourable treatment for smaller GB tumours 14. However, their lower LETs (0.2-2 keV/μm) and RBEs makes these β-emitters only efficient in case of adequate tumour oxygenation and proliferation and maybe less suitable for the treatment of radioresistant and hypoxic types of GB.

*α-particles* offer unique radiobiological characteristics, including a short tissue range (40-100 μm) and high LET, resulting in a high tumour cell-killing efficiency and corresponding RBE 155,157. Calculations have shown that as few as five high LET α-particle traversals through the cell nucleus are enough to kill a cell, whereas 10,000-20,000 low LET β-particles are needed to achieve the same biological effect 130. In addition, TAT is also suggested as a facilitator to overcome tumoral resistance to chemotherapy and the effect of radiation independently to O6-methylguanine-DNA methyltransferase promoter methylation status; the most important predictor factor in TMZ treatment 93. Of all known α-particle-emitting radionuclides, three: actinium-225, astatine-211 and bismuth-213 have received the most attention for TAT and RIT. These may be able to eradicate cerebral micro-metastases, minimally recurrent GB lesions or residual GB tumours 153,154,156.

*Auger electrons* (AE)* emitters* are characterized by an even shorter range (<100 nm) combined with a high LET and RBE. Importantly, since AE emitters are less dependent on the oxygenation state of the tumour environment, these high LET emitters could overcome the negative effects of hypoxia and necrosis 14,125,153,158-160. AE emitters might be applicable for TRT of small GB lesions but several limitations for AE-therapy may pose major obstacles for clinical translation in GB therapy. Homogenous antigen expression within the GB tumour is necessary as target-negative GB cells will potentially escape the lethal effects of AE-mediated therapy. This is a challenge for heterogeneous types of GBs especially. AE-emitting radionuclides are most efficient when incorporated into DNA. When shuttled into the vicinity of the cell nucleus where they cause direct DNA double strand breaks (DSB). Hence internalisation into the GB cells and into the nucleus is a key design aspect when considering the properties of the radionuclide combination with suitable pharmaceuticals 155. Tumour-targeted macromolecules including antibodies that bind to internalising receptors have been investigated: a locoregional administration of these favours GB cell incorporation 161,162. For example, binding of the radiopharmaceutical [^125^I]iodo-mAb-425 to the extracellular domain of the EGFR results in internalisation of the antibody-receptor complex. The specific nuclear binding of the complex then transfers iodine-125 into the cell nucleus and enables its use as a radiation source 163. Another important criterion of AE emitters is a high MA. It has a direct effect on the amount of energy delivered to a single tumour cell per receptor-recognition event and may cause a lack of essential crossfire effects 155. Although preclinical studies have shown substantial therapeutic efficacy of AE-emitters, the small number of human investigations have generally not reported clinical efficacy with the exception of some positive results with [^125^I]iodo-deoxyuridine ([^125^I]iodo-UdR) 164,165 and [^111^In]^In^-DTPA-octreotide 160,167. Treating GB patients with a [^125^I]iodo mAb 425/TMZ combination resulted in improvements of survival with minimal normal tissue toxicity, which subsequently led to the registration of a Phase III clinical trial (NCT01317888) 166,167.

### 4.2 Optimal radionuclide half-life for therapeutic application

The physical half-life of the therapeutic radionuclide should match the biological half-life of the targeted compound in order to obtain an optimal effective half-life for therapy. However, the administration route is important. When injected into the GB tumour, matching the physical and biological half-life that may be less crucial but the locoregional distribution time of the compound taken to reach the GB cells is particularly relevant. The residence-time of a radiopharmaceutical *in vivo* can be typically several days (especially with intact mAbs) or merely a few minutes for small molecules. In case of IV administration, a fast (or moderate) blood clearance capability might be more suitable as this allows for the use of radionuclides with shorter physical half-lives and minimal hematologic toxicities 111,122. However, a very short physical half-life places limits in terms of radiopharmaceutical preparation time and supply chain between preparation and injection.

**Figure 7:**
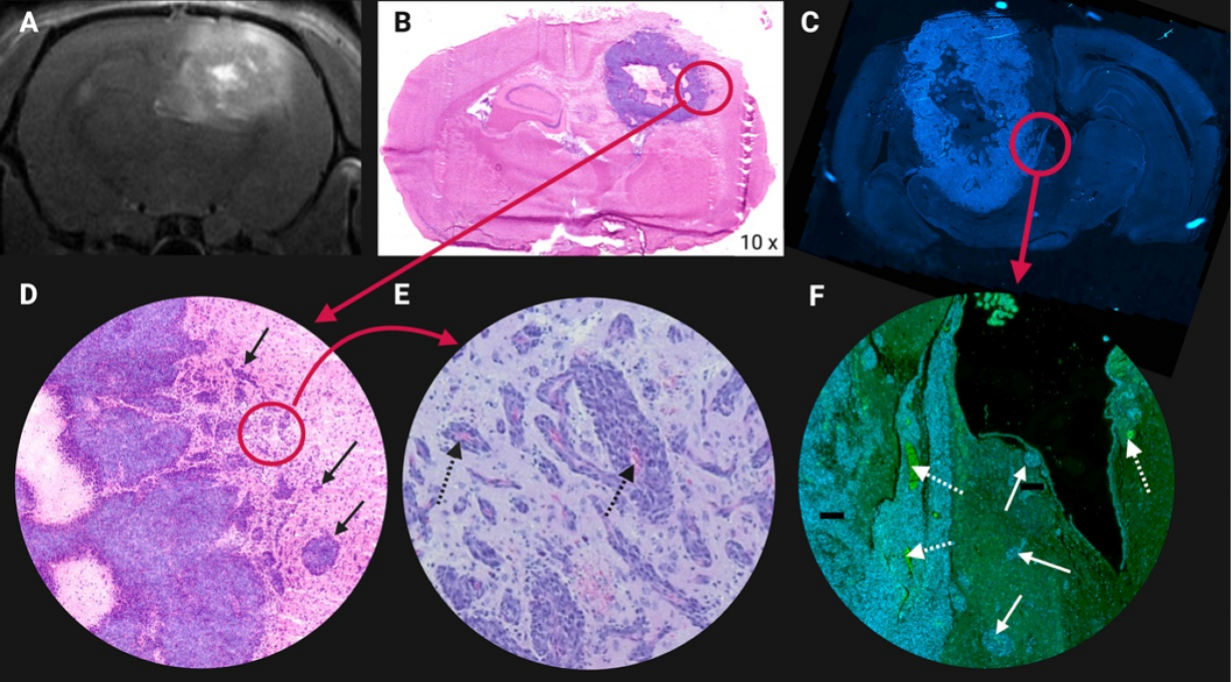
** Illustration of glioblastoma (GB) cell invasion at the tumor lesion rim in an orthotopic F98 GB rat model.** (A) Contrast enhanced T1-weighted magnetic resonance image. Higher contrast leakage in the tumour rim and in the centre of the tumour corresponds to central tumour necrosis. (B) Hematoxylin & Eosin staining. (C) 4′,6-diamidino-2-phenylindole (DAPI) nuclear staining of another F98 GB rat brain section. (D-E-F) Tumour cells infiltrating the surrounding normal brain tissue, see arrows. (E-F) Abundant blood vessels in the perinecrotic tumour, see dashed arrows. Adapted with permission from 507, copyright 2014 Journal of Neuro-Oncology.

Both the target location and the mechanism of tumoural cell uptake should match the selected radionuclide for therapy. If the target is expressed on the cell membrane, a β-emitter and a half-live of 45 min could suffice, with the prerequisite that the compound reaches the target in an appropriate time frame (to avoid multiple treatment cycles). Short-lived radionuclides might influence the uptake by infiltrating GB cells negatively, which plays a major role in GB progression and recurrence (**Figure 7**) 168. Given a compound is internalised post-binding without leakage from the target site, an AE- or α-particle emitter, providing a longer half-life e.g. up to 10 days should be considered. Negligible toxicity can only be expected if it is proven that the radionuclide is fully entrapped within intracellular macromolecular structures. In a situation where permeation out of the tumor cell can not be excluded, a high-energy, short-lived radionuclide (e.g. bismuth-213) may be recommended. In the case of AE-emitters, a longer half-life is required to provide the necessary time for its internalisation into the nucleus.

As an example, the 7.2 h half-life of astatine-211 is long enough for multistep mAb labelling procedures and is a reasonable match with the PK of intact mAbs and fragments administered in non-intravenous settings 125. Based on the information provided in this and the previous section there is no universal fit. Radionuclides for TRT with a physical half-life ranging from *six hours to seven days* to enable optimal distribution of the radiopharmaceutical in commonly large infiltrative GB tumours and to allow feasible production logistics, may be recommended 154,169.

### 4.3 Selection of a combined treatment strategy

Generally, a combined treatment strategy is suggested to advance GB treatment efficacy aim to address the following challenges: i) the infiltrative character of the tumour beyond a safety margin makes it impossible to surgically resect all GB cells, ii) systemic chemotherapy reaches the cerebral compartment only to a limited extent and iii) hypoxia and an acidotic milieu of the intratumoral and peritumoral microenvironment reduce the efficacy of EBRT and chemotherapy. Additionally, tumour heterogeneity and the multiple pathways involved could lead to signalling redundancy 93. Currently, TRT can be considered as a potent, additive treatment after the standard treatment for primary GB or as an auxiliary treatment when the tumour tissue seems to be radio- and/or chemoresistant. In case of recurrent GB, TRT could now be considered as a primary option or as salvage therapy if re-EBRT or re-chemotherapy becomes ineffective. Intracavitary RIT, in combination with EBRT, has recently been reviewed as a therapeutic strategy of high potential 106. As is the case for EBRT, TRT causes DNA damage and is therefore likely to be enhanced by combination with chemotherapeutic radiosensitisers.

Since radiopharmaceuticals (mainly peptides and mAbs) have relatively reduced drug-drug interactions, combinations of radiopharmaceuticals with chemotherapeutics may reduce interactions compared to a combination of different chemotherapeutics 135,137. Advantageously, if locally administered, no systemic side effects are caused which may increase the systemic toxicity of chemotherapy 105. Hence, TRT is now applied in combined-modality regimen 170. Basu *et al.* suggested that combining standard treatments with peptide receptor radionuclide therapy (PRRT) is attractive for patients with relatively aggressive and metastatic tumours. Monotherapy will probably be unsuccessful as inter-tumour or inter-patient heterogeneity can play a key role in many cancers, particularly in GB. Hence, therapies aiming to interfere with the protective tumour micro-environment (TME) may also use a combined strategy, pairing TRT with emerging cytotoxic agents instead of conventional chemotherapy 10,171. Other strategies might combine two synergistic TRT agents. Next to different ionizing radiation (featuring efficacy against different tumour sizes), molecular carriers with different biological properties (antibodies, peptides, organic molecules) and binding affinities to multiple tumour-associated targets are the tools to cause the desired antitumoral effects 110,170,172,173. Pre-clinically, the combination of both [^64^Cu]Cu-cyclam-RAFT-c(RGDfK)_4_ and [^64^Cu]Cu-ATSM achieved a desired anti-GB effect compared to either radiopharmaceutical because of the more uniform intratumoural distribution of radioactivity 55.

## 5. Toxicity of TRT

### 5.1 Treatment related cerebral toxicity

**Figure 8:**
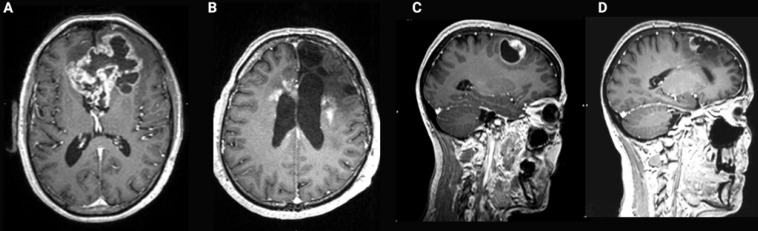
** Contrast-enhanced T1-weighted brain magnetic resonance imaging (MRI) of glioblastoma (GB).** (A) Common presentation of bulky bifrontal GB with irregular (nodular) contrast enhancement surrounding central tumor necrosis. (B) Illustration of radiation necrosis appearing as multiple foci of pathological contrast enhancement, periventricular in the left and right frontal lobe as well as anteriorly and posteriorly in the corpus callosum. (C) Nodular contrast-enhancement in a GB tumor on T1-weighted brain MRI pre-resection. (D) New irregular contrast-enhancement at the resection cavity at 1 year after a complete surgical resection reflecting tumor recurrence or treatment-related changes which have a similar appearance on MRI 31.

In current clinical practice, the treatment of GB tumours with standard EBRT is still compromised by the dose-limiting early and late toxicity to the normal brain tissue 125. Worsening cerebral edema and focal deficit are considered as early EBRT induced toxicity, while delayed toxicity symptoms may include leukoencephalopathy and cognitive decline, parkinsonism and radiation necrosis (RN). The major variables influencing the development of RN in EBRT are the radiation dose, fraction size and irradiation volume 16,174,175. Due to the localisation in a closed cavity, the risk of symptomatic increase of the intracranial pressure is high 105. Toxicity also increases with greater utilization of stereotactic radiosurgery and combined modality therapy for brain tumours 16,154,176. **Figure 8** illustrates these therapy related side effects and how they sometimes mimic a recurrent tumour on contrast-enhanced MRI 16,176,177. TRT induces toxicity during and after the treatment of GB. Its severity depends on a variety of factors covered in the following section.

#### 5.1.1 Toxicity influenced by the targeting efficiency

Toxicity to the brain is heavily dependent on the threshold of expression on a relevant target in the normal brain tissue as compared to the tumor (see **3.1.**, for crucial considerations on target selection). Ideally, the therapeutic index should be infinitely high to acquire high efficiency with minimal health risks, but in practice, this is impossible to achieve 108. However, compared to systemic chemotherapy, TRT already offers a marked improvement by allowing a tumour specific treatment. Substantial off-target distribution of the radiopharmaceutical often leads to tissue toxicity which may be widespread, with radiosensitivity the limiting factor. For example, this has been reported for the bone marrow (typically >1.5 Gy) or for lung and kidneys (1.5-2.0 Gy) 108,178. Particularly for RIT, determining parameters for an appropriate pretargeting strategy will have a great effect on the toxicity profile which would otherwise be prohibitive. However, the longevity of this therapy efficiency remains to be determined 148,149. With regard to normal tissue protection, in certain cases blocking agents can be used. For example, as both astatine and iodine belong to the halogen elements, a pre-treatment with potassium perchlorate can effectively prevent uptake of free astatine-211 and iodine-131 in cells expressing the sodium iodine symporter, e.g. in the thyroid 130.

#### 5.1.2 Toxicity influenced by radionuclide stability and the nuclear recoil effect

The stability of the radiolabelling, minimal dissociation from the targeting vector or dissociation after binding the target, are of utmost importance to prevent free radionuclides dispersing to off-target organs. This may be caused by formation of unstable complexes between the radiometal and a possibly unsuitable chelating agent. This potentially causes chemical instability, metabolism of the radiopharmaceutical or a higher affinity of the chelator for other metals resulting in transchelation and transmetallation processes 179. A crucial mechanism unique to α-emitting isotopes is the *nuclear recoil effect* causing the release of radioactive daughter nuclei (often α-emitters themselves) from the original radiopharmaceutical. This mechanism and the resulting toxicity has been reviewed in great depth in current literature 180,181. For application in GB, toxicity may be circumvented by local administration preventing the (daughter) alpha emitters to reach systemic circulation, as demonstrated for TAT using [^225^Ac]Ac-DOTAGA-SP TAT 105. Toxicity can also be prevented by internalisation of the radiopharmaceutical following binding and entrapment within GB cells 168. Although there are no nuclear recoil effects associated with astatine-211, the properties of this isotope cause unique challenges and pitfalls regarding stability as previously reviewed 182,183. Copper-64, an ideal example, circumvents toxicity associated with free radionuclide (due to instability or other sources) because free copper-64 also targets tumor tissue *in vivo* 184. Further reports investigating the recoil effect and suitable strategies to avoid its pathophysiology are anticipated.

#### 5.1.3 Toxicity influenced by physical properties of the radionuclides

When comparing TRT with EBRT, some distinct similarities exist, however, the two treatment modalities have profound differences. Like EBRT, the therapeutic index and the total absorbed dose delivered to the tumour determine the therapeutic success of TRT. Both irradiation types induce DNA damage, which leads to cell cycle arrest, DNA damage repair, cell proliferation, senescence or apoptosis. However, in GB, irradiation induced neovascularization, preferential activation of the DNA damage checkpoint and enhanced DNA repair capacity (mediated by the presence of glioma stem cells) leads to radioresistance and recurrence 185-187. Evidence is also suggesting that radiotherapy has lasting effects on the structure and composition of the GB microenvironment, facilitating tumor aggressiveness upon recurrence 188. Interestingly, combining EBRT or TRT with radiosensitizing agents could sensitize GB tumors to irradiation effects, while minimizing deleterious side effects towards surrounding normal tissues 189.

Although normally well tolerated, TRT sometimes imposes unnecessary radiation burden onto normal tissue in the vicinity of the tumour. This may occur due to the inadequate selection of radionuclide (β^-^-emitters with the highest crossfire effect) with a larger particle path length than the tumour outline would suggest 190. An important difference between EBRT and TRT is the rate at which the total dose is delivered, which impacts the biological outcome. A dose of 30 Gy delivered to a tumour over a period of many weeks at a dose rate that is exponentially decreasing, as is typically the treatment regimen with TRT, will have a very different effect from that of the same amount delivered at the much higher dose rate used in EBRT 127. It is plausible that apoptosis might be one of the mechanisms that is responsible for the higher levels of cell death at low dose rates in TRT, while others hypothesise that synchronisation in sensitive phases of cell cycle or defects in the detection of low levels of DNA damage might lie at the origin 178,191-202. In addition, radiation-induced bystander effects (RIBE) may play a significant role at low dose rates 155,178,199,202-206. It is also increasingly apparent that the paradigm of direct cell killing by the induction of DNA DSB is insufficient, since cell killing has been observed when only the cell cytoplasm was irradiated (known as non-DNA-centered effects) and in non-irradiated areas due to RIBE. In glioma cells RIBE has been shown to be mediated by nitric oxide, p53 and phosphoinositide 3-kinase. Importantly, similar signaling pathways are induced in bystander cells that are not traversed directly by α-particles 207,208. Off-target effects (e.g., bystander and abscopal effects) must be considered both at low and high doses, although it is still not known whether epidemiologically, these effects will be traduced statistically to an increase or decrease of the risk for healthy tissues 209. Interestingly, radiation may serve as a mechanism to improve the effectiveness of immunotherapy (e.g. anti-PD-L1) and change immunologically 'cold' GB tumors to 'hot' tumors by recruiting immune cells, resulting in a radiation-induced abscopal response 210. Abscopal effects of both EBRT and TRT attenuating growth of metastatic lesions elsewhere in the body is less relevant as GB is typically restricted to a single lesion (95%) within the central nervous system, with a low frequency of metastasis (0.5%) 105,211,212. Further studies are needed to validate the inverse dose-rate effect and to improve understanding of the radiobiological mechanisms involved.

#### 5.1.4 Toxicity influenced by dosimetry

The European Association of Nuclear Medicine Dosimetry Committee listed the steps required for an adequate TRT dosimetric assessment 134,213. Accurate individualised patient dosimetry with diagnostic functional imaging (SPECT/CT or preferably higher resolution PET/CT) or similar techniques are necessary to obtain an accurate risk-benefit analysis regarding normal tissue toxicity 178,214. Ideally, isotopes of the same element should be used for diagnostic imaging and therapy to improve detection of therapeutic radiopharmaceutical biodistribution (e.g. yttrium-86 for yttrium-90). TRT related dosimetric calculations must be performed for both target organs and organs-at-risk. The commonly used approach is based on the medical internal radiation dose (MIRD) formalism 215,216. More technical details on three-dimensional image-based dosimetry in TRT is described elsewhere 162,202,217-218. As individual parameters, the dimension of the cavity, the degree of radiopharmaceutical binding to the cells and the percolation into the brain-adjacent tissue were combined 222. Dosimetry using Monte-Carlo simulations also showed valuable insights for TRT of early brain metastases and concluded a preference for α-emitters 223. For very short range TRT agents such as AE emitters, it might be necessary to determine the absorbed dose at a cellular level, instead of only at the organ level 108,134,155. However, current imaging techniques do not possess the resolution required to resolve activity distributions at the microscopic or even nanoscopic scale. Hence, pre-clinical studies on cellular dosimetry and organ dosimetry using tumour xenograft models are essential 155,162. In addition, in the field of TAT, developments in microdosimetry are expanding 216,224,225.

#### 5.1.5 Toxicity influenced by immunogenicity

A specific toxicity concern in RIT is the induction of antibody immunogenicity post-administration. This elicits a human anti-mouse or human anti-chimeric antibody response, which can result in anaphylaxis or symptoms of serum sickness 135,156,226. This was noted in a phase II trial in 192 GB patients of adjuvant RIT with [^125^I]iodo-mAb 425. Four patients developed human anti-mouse antibodies preventing further administration. The development of humanized and fully human mAbs could prevent this immunogenic response 155,227. The avidin-biotin pretargeting system in GB has also shown to induce high immunogenicity of streptavidin in almost all patients (90%) 147,148. Small peptides (<4 kDa) are generally believed to be poor immunogens, despite some exceptions being observed 135. To limit immunogenicity (preferable a LD_50_ > 1.5 g per kg of body weight), small molecules and peptides are preferable to mAbs. The design of these radiopharmaceuticals should involve strategies to reduce immunogenicity such as avoiding the inclusion of antigenic amino acid sequences and employing structural modifications, such as glycosylation or PEGylation, which tend to shield antigenic determinants from detection by the immune system 135,154.

#### 5.1.6 Toxicity specifically associated with the CED tumour administration route

In CED, the therapeutic agent is delivered directly into the tumor which imposes a significant concentration differential across the tumour boundary dependant on leakage into the surrounding tissue, thereby minimising systemic toxicity and neurotoxicity. Local injection also minimizes renal risk from potential tubular re-uptake of the radiopharmaceutical 105,228. Inflammation adjacent to the catheter tract and at the catheter tip is shown to be limited to within a 50 mm radius and CED does not produce cerebral edema or any measurable increase in intracranial pressure 128,129. However, increased interstitial fluid pressure within the brain tumour can drive the infusate into relatively low-pressure areas in surrounding normal tissues. Furthermore, catheter-induced tissue damage can occur and backflow may be significant in cortical infusions, leading to subsequent widespread distribution of the agent within the subarachnoid space. The latter can also be induced by leakage from the postsurgical cavity to cerebrospinal fluid spaces, in the event of a connection, which is a major contraindication for TRT. It can lead to an inflammatory reaction of the brain, a diminished concentration of the radiopharmaceutical within the tumour and an increased risk for widespread neurotoxicity 105,128. An adequate stereotactic positioning of catheters and a careful application of the compound is of utmost importance. Co-injection of the imaging counterpart together with the therapeutic dose allows short time imaging of the tumor and study of the whole body distribution, and is recommended for monitoring adequate distribution 93.

### 5.2 Clinical toxicity resulting from TRT of GB

In general, current clinical results show that newly diagnosed and recurrent brain tumor patients who have been treated with TRT often show only limited adverse effects. It should be noted that not all clinical trials contain plausible evidence on clinical toxicity. The most relevant examples for each radioisotope are described in the following paragraphs.

#### 5.2.1 Iodine-131

In phase I/II trials including diverse malignant gliomas different iodine-131-labelled tenascin-mAbs were injected directly into the tumor or the resection cavity, resulting in minimal toxicity 96,126,229-233. Systemic and neurological toxicity were negligible in 10 recurrent GB patients receiving doses ranging from 111-1147 MBq per cycle [^131^I]iodo-BC-2 stereotaxically 231. Similarly, in 30 recurrent GB patients, a higher intratumoral dose of 1100 MBq [^131^I]iodo-BC-4 did not result in adverse systemic effects 236. This approach was confirmed in a large phase I/II clinical trial including 111 patients who suffered diverse malignant gliomas. For the phase II component, patients received a mean dose of 1.29-2.78 GBq with minimal toxicity 233. In another phase II trial, 43 patients with recurrent malignant glioma (GB: n=33), 3.7 GBq of [^131^I]iodo-m81C6 was injected directly into the surgically created resection cavity (SCRC) followed by chemotherapy with acceptable tolerability and toxicity. Acute, primarily reversible, hematologic toxicity was the most common significant adverse event (23%). In 12% of the population acute neurotoxicity developed but this resolved spontaneously or after short-term corticosteroid administration in all except one patient 96. The maximum-tolerated dose of [^131^I]iodo-m81C6 into the SCRC was 4.44 GBq in a phase I trial which involved 42 malignant glioma patients with no prior EBRT or chemotherapy 232. A dosimetric study did not detect neurological toxicity while minimal hematologic toxicity occurred with the maximum tolerated administration of 3.7 GBq [^131^I]iodo-m81C6 95. Akabani *et al*. in 2005 suggested an optimal absorbed dose of 44 Gy to the 2 cm cavity margins to reduce the incidence of neurologic toxicity 234.

In 2008, a targeted 44 Gy boost of [^131^I]iodo-m81C6 was delivered to the SCRC followed by EBRT and chemotherapy in 21 newly diagnosed malignant glioma patients (GB, n=16), which was well tolerated and had an encouraging survival outcome 235. The dosing regimen of an [^131^I]iodo-chTNT-1/B mAb targeting DNA histone H1 complex (Cotara^®^) was determined to be 37.0 to 55.5 MBq/cm^3^ without toxicity 236-238. In a cohort of 51 patients with histologically confirmed malignant glioma (GB n=45) which received Cotara via CED and the treatment-emergent, drug-related CNS adverse events included brain edema (16%), hemiparesis (14%), and headache (14%). These events were mostly reversed with corticosteroid co-treatment. Systemic adverse events were predominantly mild 238. Intracavitary-administered [^131^I]iodo-TM-601, a recombinant version of chlorotoxin, was well tolerated, without dose-limiting toxicities or clinically significant acute adverse events during infusion of [^131^I]iodo-TM-601 at any dosage being observed during the 22-day observation period. Grade 3 or 4 toxicities related to the study drug or method of administration were not observed in the immediate or long-term follow-up periods 239. In a human trial of systemic endo radio-therapy with [^131^I]iodo-IPA (up to 6.6 GBq), patients did not present with acute or late radiotoxicity, neurotoxicity, and haematological or renal adverse events were not observed. This first-in-human investigation was performed in two patients with progressive gliomas, which were initially diagnosed as low-grade astrocytoma (WHO II) and oligodendroglioma (WHO II), respectively 240.

#### 5.2.2 Yttrium-90

Adverse events remained well controllable with the fractionated dosage regimen of [^131^I]iodo- or [^90^Y]Y-anti-tenascin mAb applied in 55 malignant glioma patients (GB n=40) 229. In 73 recurrent GB patients treated with the “3 step” [^90^Y]Y-biotin based loco-regional RIT, safety and efficacy was also shown 149. [^90^Y]Y-DOTAGA-SP was locally administered into the tumours of 14 pilot study patients except for critically located tumours which were injected with [^177^Lu]Lu-DOTAGA-SP and [^213^Bi]Bi-DOTAGA-SP instead. Drug-related toxicity did not present but disease stabilisation or improved neurologic status was observed in 13 of the 20 patients while neurological function improved in 5 out of 14 GB patients within 2 weeks 241. In a prospective phase I study involving 17 GB patients, [^90^Y]Y-DOTAGA-SP treatment was well tolerated by all patients without acute toxicity or other side effects 247. Of 43 GB patients treated with 0.4 to 3.7 GBq of [^90^Y]Y-DOTA-lanreotide using a fractionated 1- 6 therapy cycle, disease regression and a subjective improvement in quality of life measures was reported in 5 patients while 14 patients presented with stablised disease 62. When [^90^Y]Y-DOTA-TOC was administered to 3 GB cases in three or four fractions at intervals of 3 to 4 months (1.7 to 2.2 GBq), the only observed adverse effects were a reoccurrence of an epileptic seizure for one patient and a mild transient headache for another. In general all patients reported an improved quality of life 59. In an extended pilot study by Schumacher *et al*., 10 low-grade and anaplastic glioma patients received local administration of varying fractions of [^90^Y]Y-DOTA-TOC, either into the tumour or the resection cavity without associated intermediate- to long-term toxicity 60.

#### 5.2.3 Rhenium-188

The radiolabelled anti-EGFR ligand [^188^Re]Re-nimotuzumab was administered intracavitary to 3 patients with anaplastic astrocytoma and 8 GB patients in an open, uncontrolled, dose-escalation phase I clinical trial. In patients treated with 370 MBq (n=6) transitory worsening of pre-existing neurological symptoms was observed. Two patients treated with 555 MBq (n=4) developed early severe neurological symptoms and one patient also developed late severe toxicity involving RN. Single doses of [^188^Re]Re-nimotuzumab were also locoregionally administered to 9 recurrent GB patients with a maximum tolerated dose of 370 MBq 243.

#### 5.2.4 Lutetium-177

A progressive pontine GB case, pretreated with EBRT and TMZ, [^177^Lu]Lu-DOTAGA-SP (1.13 GBq) was injected via a transcerebellar catheter without side effects. Clinical and radiologic improvement lasted for 5 months. It is of note that two more cases within the same study (but presenting with oligoastrocytoma and astrocytoma, WHO grade III) received higher doses of [^177^Lu]Lu-DOTAGA-SP (2.25 and 6.38 GBq). Impaired neurologic function markedly improved significantly in both within 2 weeks after injection. However, intermediate or long-term toxicity could not be evaluated in a patient who died following tumor progression 241.

#### 5.2.5 Astatine-211

Anti-tenascin 81C6 mAb was also labelled with astatine-211 (71-347 MBq) and injected in the surgically created resection cavity of 18 recurrent malignant brain tumors (GB, n=14). While dose-limiting toxicity did not occur, 6 patients experienced reversible grade 2 neurotoxicity, and the median survival time for GB patients was 52 weeks. This compares favourably to 23-31 weeks for patients receiving conventional therapies 244.

#### 5.2.6 Bismuth-213

In a pilot study 5 patients with critically located gliomas (WHO grades II-IV) were locally injected with [^213^Bi]Bi-DOTA-SP. Treatment was tolerated without additional neurological deficit or local or systemic toxicity 242. In a case presenting with progressive GB, intracavitary injection of 375 MBq of [^213^Bi]Bi-DOTAGA-SP was tolerated well 241. Nine recurrent GB patients received 1 - 6 intracavitary doses of [^213^Bi]Bi-DOTA-SP in 2-month intervals (median 5.8 GBq), which was well tolerated with mild transient adverse reactions (mainly headaches caused by transient perifocal edema) 114. In a more recent trial treatment with activity up to 11.2 GBq [^213^Bi]Bi-DOTA-SP was well tolerated in 20 patients with recurrent GB with mild and transient adverse reactions (edema, epileptic seizures, aphasia) 93,105. During [^213^Bi]Bi-DOTA-SP infusion, facial erythema was observed in a few patients: a systemic effect caused by a small amount of [^213^Bi]Bi-DOTA-SP absorbed into the blood 105.

#### 5.2.7 Actinium-225

Local administration of [^225^Ac]Ac-DOTAGA-SP was well tolerated, with mild, transient observations of edema, aphasia or epileptic seizures 93,105. In one patient with the tumour located in the left temporal lobe, injection of the [^225^Ac]Ac-DOTAGA-SP induced hemiparesis and hemianopia 3 days later, lasting several months 105.

#### 5.2.8 Iodine-125

In a phase II clinical trial [^125^I]iodo-mAb 425 was administered intravenously or intra-arterially as an adjuvant therapy in 118 GB patients (mean dose of 5.2 GBq). Acute and chronic toxicity presented as an exception in 1 of the 118 patients as hypothyroidism 166. In a second phase II clinical trial of 192 GB patients subjected to surgery and EBRT followed by 3 weekly intraveneous injections of 1.8 GBq, grade 3/4 toxicological events did not occur 227.

## 6. Validating new radiopharmaceuticals

The transition of radiopharmaceutical therapeutics to a clinical setting have been extensively reviewed, including therapeutic radionuclide production, preclinical evaluations and Good Manufacturing Practice (GMP) perspectives; however, these mostly lack information that would address GB specifically 245,246. Current regulatory status and broad guidelines regarding the clinical translation of radiopharmaceuticals, with particular emphasis to the European context, has also been reviewed 247-249. Further guidance is anticipated specifically regarding radiopharmaceuticals targeting GB, necessary because of the complex radiobiological considerations they entail. Thus, the need for monographs for therapeutic products is urgent and standardisation of quality control and assurance procedures are of utmost importance. A major limitation of radiopharmaceutical commercialisation is meeting product demand and special requirements of the market in nuclear medicine adequately. An excellent example of the translation to the clinical setting is the report on Lutathera^®^ ([^177^Lu]Lu-DOTA-TATE), describing its product characteristics, quality control procedures with an application guide 57. In this section the relevant knowledge, technicalities and major factors influencing the validation of radiopharmaceuticals, especially for prospective GB imaging and therapy will be discussed.

### 6.1 Target-based selection of compound candidates

Since the physiology and pathology of GB tumours is so unique, it is important to use complimentary techniques to increase the probability of accurate characterisation. After identifying a promising GB target (Section** 3.1**), exploration of target specific compound candidates involves stringent *in vitro* investigations. Carager *et al*. discusses most recent systems for *in vitro* brain cancer research but those selected are defined by the targeted GB pathophysiology 250. Importantly, the advent of* in vitro* three-dimensional GB colloid models include a better representation of *in vivo* cell environments (eg. hypoxic cell status) which could result in more accurate predictions of efficacy and sensitivity before any *in vivo* investigations are launched 251-253. Central necrosis may occupy as much as 80% of the total GB tumor mass and includes “dormant” hypoxic tumor cells that may be very radioresistant 189,254. In addition, the use of an *in silico* molecular modelling step addressing the design of radiopharmaceuticals for GB is strongly recommended. For the selection process of the optimal complexation strategy for radiometal-complexing bioconjugates, it should be noted that available published data, often published concerning the non-medicinal isotopes of a certain radiometal (available from radioassays for *in vitro* investigations), are not applicable as input for further compound candidate selection. Designing radiopharmaceuticals by adaptation of naturally-occurring bioactive molecules, conventional drug candidates, or established molecular imaging probes remains a sound approach. That being said, however, the timelines and success rates may potentially be improved by incorporating proteomics, genomics and computational methods in the design process of new candidates 255,256. Elegant study examples are the *in silico* modelling of antibody immunogenicity potential and the calculation of radiobiological mechanisms applied to cancer cells for translation to bulky tumours 81,257. Interestingly, Rockne *et al.* suggest the creation of a virtual *in silico* tumour with the same growth kinetics as that in a particular patient to predict efficacy based on *in vitro* responses. Although this study looked at the response to RT, translating this concept to radiopharmaceutical design is plausible 258. *In silico* modelling offers the means of combining both *in vitro* data and computational power to create intricate pharmacokinetic-pharmacodynamic modelling to facilitate the design process and potential to improve therapeutic outcome. Therefore it should be incorporated into the theranostic protocol 259.

### 6.2 Radiosynthesis requirements

A variety of strategies and optimised protocols for efficient labelling of peptides, mAbs and other targeting vectors have been published but specific details to develop GB-specific radiopharmaceuticals are scarce 259-262. The vast application of radio-metal isotopes emphasises the intricate nature of complexation chemistry in GB therapy. When considering metal therapeutic radionuclides, the choice of chelators to be incorporated in the radiopharmaceutical to yield the most stable complex is crucial for recommending a metal-radioisotope as appropriate (**Table 2**) 263-266. If certain *in vitro* applications to characterize a radiopharmaceutical are preferred, it is important to meet the stipulated criteria qualifying their use, when performing these tests on cells or tissue. For example, the radioligand association constants obtained from any chelator-ligand pair (without any radioisotope) can already differ markedly from its radioisotope-chelator-ligand complex derived from radiosynthesis (also relevant to its formulation). In addition, free radionuclides *in vivo*, either from lack of complexation integrity or poor labelling, can lead to unfavourable organ toxicity. If radiopharmaceutical instability (*in vivo* or benchtop) is an issue, multiple strategies for stabilization thereof have been reviewed 267. For instance, it is considered good practice to add diethylenetriamine pentaacetate (DTPA) after radiolabelling some metallic based therapeutic radiopharmaceuticals to chelate any uncomplexed radionuclides.

The chemical considerations of astatine-211 and iodine-131 as therapeutic halogens are unique with different constraints 182,268-270. Production and isolation of astatine-211 is well described and feasible but complete radiopharmaceutical production infrastructure is less widely spread. Lindegeren *et al.* describes the inherent intricacies involved and provides insights into how the chemistry infrastructure could be developed 269. One of the most important factors to consider is the MA of the final radiopharmaceutical. If the production methods introduce carrier molecules into the formulation (e.g. carrier-added lutetium-177) then the targeting vectors labelled with non-radioactive nuclides (such as lutetium-176) may compete with target binding reducing uptake of the radiopharmaceutical. An example of a sophisticated system is to use uncomplexed copper-64 dichloride produced with high specific activity for GB without additional influences on MA. Optimal internalisation can take place at the target through the copper transporter in GB 184.

### 6.3 Quality control validation

Since therapeutic radiopharmaceuticals have unique characteristics including different types of emissions and half-lives, quality control can be challenging. The methods used are often spairingly applied to therapeutic radiopharmaceuticals in comparison to diagnostic radiopharmaceuticals. The longer half-lives of some therapeutic radionuclides complicates the common procedures used for quality control, i.e. sterility testing and testing for pyrogens. Therefore, this requires additional infrastructure investment for “in-house” quality control. The emission characteristics of α-emitters and pure β-emitters complicates high performance liquid chromatography (HPLC) analysis, but it is strongly recommended that the quality control is not only performed by unvalidated instant thin-layer chromatography (ITLC) methods. Subtle changes in stability of the vectors and effects of radiolysis would not necessarily manifest clearly using ITLC analysis. Regarding therapeutic peptides, it is important to not only focus on radiochemical stability but also take into account subtle changes in peptide structure (causing chemical instability) that could be brought about by radiolysis. Importantly, certain α-particle emitting radionuclides may necessitate special QC requirements. For example, since actinium-225 does not emit γ-rays, a delay of 60 min is necessary after ITLC to obtain radiochemical equilibrium between actinium-225 and its daughter nuclide francium-221 271,272. Once the radiosynthesis parameters and quality control techniques are established, automation of radiopharmaceutical production can be of considerable support achieving robust, GMP compliant products for clinical trials and providing proven validated radiation protection for operators 273-275.

### 6.4* In vivo* validation using GB models

Rodent GB tumour models which have been the main research tools of preclinical investigations for over 30 years may be subdivided into three categories: ethyl-nitrosourea (ENU) -induced gliomas, genetically engineered models (GEMMs) and patient-derived xenograft or glioma cell models (PDX or PDGC); as summarised in **Table 3** 276-278. *In vivo* validation of novel theranostic compounds should include *in vivo* studies on biodistribution, tumour uptake, therapy monitoring and toxicity 279. Particularly important for GB is determination of the optimal administration method by comparing intraveneous administration (BBB passage testing) with CED or intra-tumoral injection. The distribution and the residence time of the targeting compound throughout the GB tumour and in healthy brain tissue should be analysed comparatively. In cognisance of this, efficient retention via CED in GB models has been reported 165,280,281. The efflux effect of the selected radiopharmaceutical at the BBB can be studied in biodistribution experiments or PET imaging while co-injecting a P-gp blocker (eg. tariquidar or loperamide) 47. In addition, PET biomarkers have been designed to image (and quantify) these undesired efflux transporters present on the BBB 282. Overall survival analysis and therapy follow-up using PET/SPECT/MRI imaging should be performed to test the efficacy of single and fractionated doses, including an estimation of the delivered therapeutic dose. Multidrug approaches, including the currently used first-line chemotherapeutic agent TMZ should be included. Auxiliary tests to study dose-limiting and off target effects would require blood/urine/faeces sample collection and post-mortem histology.

As highlighted by Lenting *et al*., valid brain tumour models should fulfill critical needs to yield relevant data for the prospective success of radiopharmaceuticals in Nuclear Medicine 276,283. For therapy studies, the tumors should be either non- or weakly immunogenic in syngeneic hosts. Subcutaneously (heterotopic) GB tumour models are technically simple and enable rapid determination of treatment efficacy. However, these result in encapsulated, non-invasive tumours and caution is advised when therapeutic activity focuses on disturbing the interaction with the TME 276,279,284,285. Orthotopic GB models include a CNS micro-environment and would be more appropriate to test the BBB passage of a new TRT compound 284. In case of a RIT agent, preclinical testing with the currently available glioma tumour models remains outstanding. As the immune-compromised status of the recipient mice renders PDXs inadequate for this purpose, humanised mice are required. In addition, the use of PDX models is often hampered by donor availability and limited propagation 277. ENU-induced gliomas recapitulate human gliomas most faithfully with respect to genetic heterogeneity and immunocompetence, but often lack reproducibility 276. As expected, each of the listed animal models has its advantages and limitations. An emerging alternative is represented by the organoids generated from human samples and so-called organoid-derived GB xenografts, including the generated live biobank established by Jacob *et al.* 10,286-288. Hence, the TRT efficacy should be tested in a combination of suitable models to compile sufficient information for optimal design of the clinical protocol.

### 6.5 Data required for clinical translation

**Figure 9:**
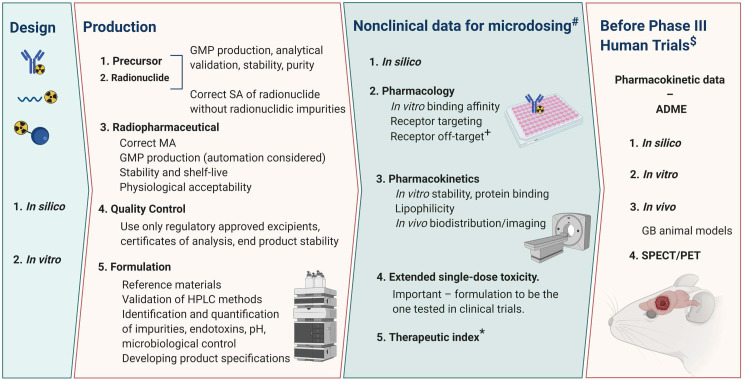
** Quality data required for translation of a radiopharmaceutical.** The sequential approach to an adequate validation of radiopharmaceuticals is illustrated; certain tests and validation steps may not depend on each other and are therefore often performed in parallel. Abbreviated and footnoted content: Absorption Distribution Metabolism Excretion (ADME), good manufacturing practice (GMP), molar activity (MA), specific activity (SA), glioblastoma (GB), positron emission tomography (PET), single-photon emission computed tomography (SPECT). ($) i.e.: target validation, (*) a requirement only for the validation of therapeutic radiopharmaceuticals, (#) not required for microdosing e.g. radiopharmaceuticals (<100 µg); e.g. genotoxicity, safety pharmacology, repeat dose toxicity. (+) radiolabelling may alter the pharmacological characterisation of the targeting molecule; pharmacological effects should be ruled out at the anticipated clinical dose 247.

Before a new radiopharmaceutical may be introduced in the clinic, a range of assessments (**Figure 9**) are required 245,247,289. An investigational medicinal product dossier (IMPD) is required for regulatory boards. Information regarding the IMPD requirements in the USA and Europe pertinent to radiopharmaceuticals is available, but it is important to take into account regional requirements and to work with local governing bodies 249,290,291. If the theranostic partnership contains two separate radiopharmaceuticals (e.g. [^68^Ga]Ga-DOTA-TATE and [^177^Lu]Lu-DOTA-TATE), two seperate radiopharmaceutical production validations must be performed. A significant amount of information concerning the release criteria, analytical procedures and their validation must be provided 247. Most importantly, validation ensures a robust method (taking into account operator variability) and focuses on reproducibility of manufacture and quality of the environment. Following product validation, nonclinical (*in vitro* and *in vivo*) safety data (**Figure 9**) is imperative. It is of significance that FDA guidelines require that, for diagnostic procedures, risks are low and the associated translation requires much less *in vivo* valuation 292,293. However, caution is advised as assumptions regarding radiopharmaceutical safety based on experience of diagnostic radiopharmaceuticals may be inaccurate if applied to therapeutic equivalents. These therapeutic radiopharmaceuticals may demonstrate inherent additional toxicity (often connected to off target effects). Their *in vivo* stability can be more critical; they are also often injected in repeat doses in shorter time frames than required for diagnostics). Consequently, additional tests (selectivity, pharmacokinetics, sensitivity and safety) beyond those usually required for diagnostic radiopharmaceuticals are included by the validation process. In particular for GB, the* in silico* calculations of radiation dosimetry and the toxicity profiling may become more complex and must be performed stringently.

## 7. Future perspective

Prospective strategies for a multi-targeted approach may include the use of heterobivalent or hetero-multivalent ligands which may bind simultaneously or monovalently to their different molecular targets. This is supported by the observation of Reubi *et al.* that non-endocrine tumors (including GB) concomitantly express several peptide receptors at a high density 294. Considering that strategies successfully reversing GB hypoxia are likely to improve the response to radiation significantly, a prospective treatment strategy is proposed. This entails the administration of a tumour tissue penetrating *hypoxia-promoting TRT* agent to treat the centrally located tumour region in parallel with the administration of an anti-proliferating TRT agent to target the viable tumour boundaries adequately. As a higher LET radiation is less dependent on the oxygen enhancement ratio, α-particle and AE emitters might be more effective in targeting hypoxic regions. In addition, targeting with high LET irradiation initially may be the best option to tackle the most radio-refractory cells from the very beginning of treatment because of the characteristic rapid progression of GB.

**Figure 10:**
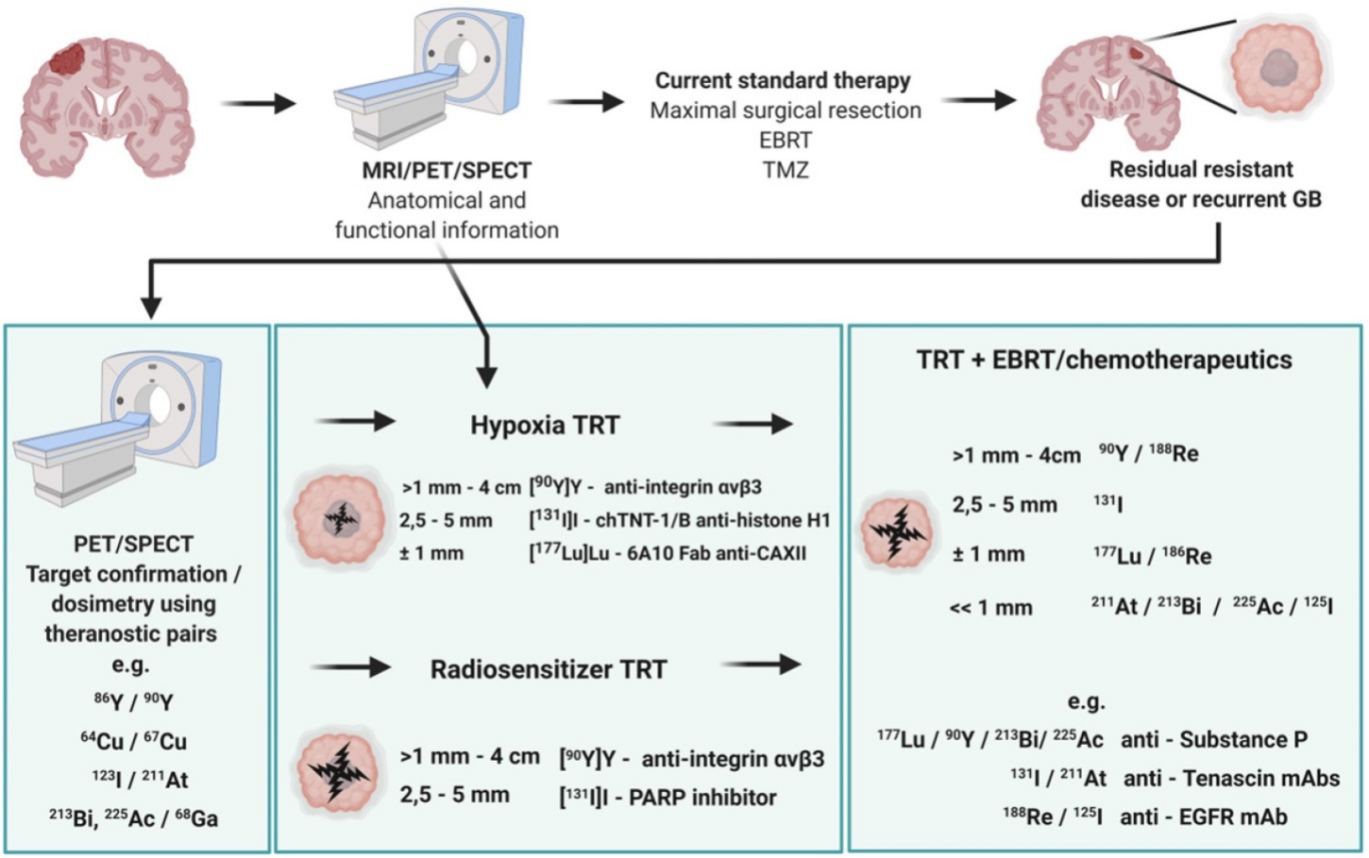
** Future scenario: combined targeted radionuclide therapy of glioblastoma tumours.** The prospective glioblastoma management will include practicing of various combinations of therapeutic tools. Abbreviated content: glioblastoma (GB), targeted radionuclide therapy (TRT), temozolomide (TMZ), external beam radiotherapy (EBRT), magnetic resonance imaging (MRI), positron emission tomography (PET), single-photon emission computed tomography (SPECT).

Strategic combined administration of an ^211^At-labelled compound in tandem with an ^131^I-labeled compound to maximise dose deposition in residual tumour margins, is expected to be successful 125. A ^131^I-labeled chimeric mAb ([^131^I]iodo-chTNT-1/B; Cotara^®^) is a valid first TRT option for patients presenting with largelly hypoxic GB, as its target (histone H1 complexed to DNA) is abundantly present within the necrotic core of GB tumours 111,237,238. Possibly more relevant in future, a different radionuclide cocktail for GB treatment is RGD-based integrin antagonists radiolabeled with either lutetium-177 or yttrium-90. Hypoxia may trigger the recruitment of αvβ3 integrins to the cellular membrane of such conditioned GB cells. Blocking αvβ3 integrins with RGD reduces the intracellular levels of the hypoxia-inducible factor 1α 300. Besides targeting hypoxia, TRT compounds that directly bind to targets expressed in necrotic or apoptotic cells as part of the GB core may be recommended. However, the available data is limited despite the development of imaging biomarkers, such as [^18^F]F-pyrophosphate, [^18^F]F-glucaric acid, [^99m^Tc]Tc-Annexin-V and [^18^F]F-2-(5-fluoropentyl)-2-methyl malonic acid 301-303. Radiation- and chemo-resistance are major obstacles in GB treatment and add another concept to be explored: *radiosensitizer radiopharmaceuticals (RR).* Low-LET radiation may be potentiated by inhibitors of DNA damage repair or disruptors of cell cycle control. Radionuclides with high-LET might be optimally combined with radiosensitizers that do not depend on the generation of reactive oxygen species 170. Hence, RR targeting DNA repair pathways, cell cycle progression or growth factors could be administered first to enhance the cytotoxicity of subsequently administered ionizing radiation. Different types of radiosensitizers, including small molecules, macromolecules and nanomaterials, were recently reviewed 299. An elegant example is the administration of a ^131^I-labelled PARP inhibitor, which was recently tested in a GB animal model *in vivo,* but its therapeutic efficacy still needs to be confirmed 280,299. **Figure 10** suggests a few recommendations for possible future TRT (combined) treatment strategies for GB.

Alongside the promising future perspectives of TRT, other radiation-based treatment options are likely to bring additional new developments for future GB therapy. Radiosurgery, brachytherapy (BT), a new method of BT, termed diffusing α-emitters RT (DaRT), and boron neutron capture therapy (BNCT) have been explored for GB management with varying outcomes 301-305. A new avenue that diminishes normal tissue toxicity whilst maintaining an equivalent tumour response is the development of ultra-high dose rate (FLASH) RT. In FLASH RT, the dose is delivered at ≥ 40 Gy/sec compared to dose rates of approximately 1-4 Gy/min in conventional EBRT 306. This technique provided encouraging results in an *in vivo* study using a murine GB model but is currently still limited to superficial tumors using electron beams 307.

## 8. Summary and Outlook

TRT can be considered as an adjuvant treatment to the standard treatment for primary or recurrent GB or as a secondary treatment when the tumor tissue is radio- and/or chemo-refractory. For recurrent GB all current treatment interventions are only given with a palliative intent. In these cases, TRT and biomarkers/imaging (MRI/PET/SPECT) might offer new possibilities for individualised treatment based on a combination of clinical findings, the genetic and molecular profile of the patient in relation to his/ her GB pathology. Such advanced molecular imaging enables, for example, the calculation of optimal dosage to achieve maximal treatment response with minimal toxicity and to prevent over-treatment 308.

**Figure 11:**
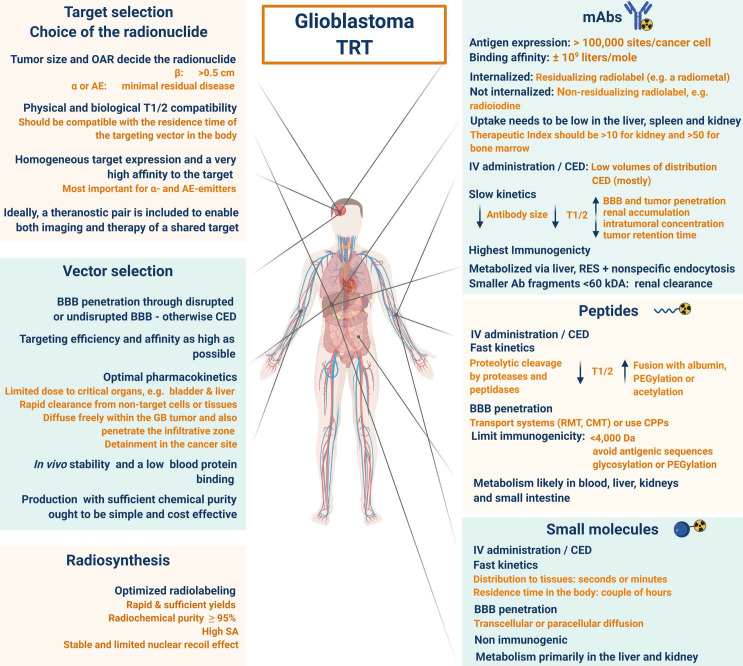
**Summary of targeted radionuclide therapy of glioblastoma.** Main consideration for the development of new radiopharmaceuticals for brain tumours, comparing three different radiolabelled vectors (small molecules, peptides and monoclonal antibodies (mAbs). Abbreviations and footnoted contents: Auger electron (AE), blood-brain barrier (BBB), convection-enhanced delivery (CED), cell-penetrating peptides (CPP), intravenous (IV), organs at risk (OAR), reticulo-endothelial system (RES), receptor-mediated transport (RMT), specific activity (SA), targeted radionuclide therapy (TRT), carrier-mediated transport (CMT), half-life (T1/2).

Given the fact that recurrence of GB is probably inevitable, TRT could be more effective if given immediately after standard therapy or immediately after diagnosis of recurrence, depending on the clinical state of the patient 105. Clinical reports supporting the outcome of the latter principles are expected to emerge shortly. The major types of TRT that are being explored for GB therapy include PRRT, ligand based radionuclide therapy and RIT. Main considerations for the development of new radiopharmaceuticals for brain tumours are summarised in **Figure 11**, utilizing the three most radiolabelled vectors.

Isotope availability, the parameters of radiosynthesis and off-target toxicity are significant limitations to achieving improved process standardization. Two major requisites for successful TRT of GB are evident: i) a well differentiated tumour that expresses the desired target in ample quantities without normal physiological target functions and ii) highly specific ligands with high molar activity that are able to overcome biological barriers. BBB crossing (often disrupted by GB), tumour diffusion, internalisation and intracellular accumulation are significantly affected by the vector design irrespective of the selected optimal radionucleotide. Additionally, strategies to enhance BBB crossing or CED administrations need to be considered to increase TRT effects.

It is imperative that proper consideration may be given to large-scale production of radionuclides for TRT, with its organisation in an economic and GMP-compliant manner. Scarcely available radionuclides or those with an expensive production infrastructure, despite having attractive characteristics, are unlikely to be used routinely 154.

A limited number of clinical studies using AE emitters as cancer therapy tools have been performed 155. In GB patients, anti-EGFR [^125^I]-Iodo-mAb 425 did show promising results 227. Available α-particle emitters, with their short range and high LET and RBE seem appropriate for GB therapy as they may minimize harm to surrounding healthy brain tissue thereby triggering high cell kill-rates, with minimal dependency on cell cycle and oxygenation status 168. In contrast, the longer range and cross-fire effect of β-emitters engender them to a more heterogeneous target distribution 10. Further research on inverse dose rate effects that may affect the absorbed dose-effect relationship in TRT should be the focus of future preclinical studies in radiobiology. Besides performing accurate dosimetry, the most relevant biological endpoints must also be identified. Current clinical results show that brain tumor patients who have been treated with all three types of therapeutic radionuclides generally show limited adverse effects. A combined treatment strategy may produce more effective outcomes by targeting multiple pathways critical for cancer progression. Optimally (randomised, multi-centered) controlled trials are urgently needed to establish the ideal management strategy for GB, in particular concerning AE emitters, combining radiopharmaceuticals and demonstrating its alliance with other systemic therapies, such as immunotherapy 178.

## Figures and Tables

**Table 1:** Investigational PET/SPECT imaging in neuro-oncology

Biological target		Radiopharmaceuticals^($)^	Vector^(ǂ)^	References
Amino acid metabolism	C	[^18^F]F-ACBC [^18^F]F-tryptophan [^18^F]F-Glutamine [^18^F]F-FSPG [^123^I]iodo-IMT [^123^I]iodo-IPA	AA AA AA AA AA AA	49,51,311-317 318-323 324 325 326 326-328
	P	[^18^F]F-ELP [^18^F]F-AMPe [^18^F]F-A(M)Hep [^11^C]-/ [^18^F]F-tryptophan [^18^F]F-Glutamine [^18^F]F-IMP	AA AA AA AA AA AA	329,333 331 332 333-335 336,337 338
Angiogenesis (Integrin receptor family)	C	[^18^F]F-/ [^68^Ga]Ga-PRGD2	Pep	53,339-342
	P	[^64^Cu]Cu-PEG_4_-c(RGDyK) [^68^Ga]Ga-c(GDGEAyK) [^111^In]In-abegrin™ [^99m^Tc]Tc-NC100692 [^18^F]F-fluciclatide [^18^F]F-PPRGD2 [^18^F]F-RGD-K5/ [^68^Ga]Ga-RGD [^64^Cu]Cu-c(RGDfK)]_2_ [^64^Cu]Cu-c(RGDfK)_4_ [^64^Cu]Cu-PEG_4_-E[PEG_4_-c(RGDfK)]_2_ [^64^Cu]Cu-Gly_3_-E[Gly_3_-c(RGDfK)]_2_ [^18^F]F-alfatide II	Pep Pep Ab Pep Pep Pep Pep Pep Pep Pep Pep Pep	343 344 345 346 347 348 349 350 54 351 351 352
Angiogenesis (Vascular endothelial growth factor receptor)	C	[^123^I]iodo-VEGF-165	Prot	353
	P	[^111^In]In-ZVEGFR2-Bp2 [^89^Zr]Zr-bevacizumab [^64^Cu]Cu-VEGF121 [^64^Cu]Cu-VEGF125-136 [^111^In]In-hnTf-VEGF	Abf Ab Prot Pep Pro	354 355 72,74 356 357
Epidermal growth factor receptor	C	[^11^C]-CPD153035	SM	358
	P	[^124^I]/ [^131^I]iodo-IPQA [^11^C]-/ [^18^F]F-ML01/-03/-04 [^64^Cu]Cu-/ [^111^In]In-cetuximab [^111^In]In-/ [^125^I]iodo-ch806 [^18^F]F-BEM-/ [^68^Ga]Ga-ZEGFR:1907 [^89^Zr]Zr-nimotuzumab [^188^Re]Re-U2 (ç) [^18^F]F-B-ME07 (°) [^111^In]In-hEGF	SM SM Ab Ab Abf Ab ON ON Ab	359 360 361,362 363,364 365 366 367 368 369
Chemokine receptor 4	C	[^68^Ga]Ga-pentixafor	Pep	94
	P	[^11^C]methyl-AMD3465	SM	370
Ephrin receptors	C	[^89^Zr]Zr-ifabotuzumab	Ab	371
	P	[^64^Cu]Cu-IIIA4 [^64^Cu]Cu-TNYL-RAW [^64^Cu]Cu-1C1	Ab Pep Ab	77 40 362
Hypoxia	C	[^18^F]F-DiFA [^62^Cu]/ [^64^Cu]Cu-ATSM [^18^F]F-EETNIM	SM SM SM	372 373-375 376
	P	[^18^F]F-RP170 [^18^F]F-HX4 [^62^Cu]/ [^64^Cu]Cu-ATSM	SM SM SM	377 378 55,67,75,379
Poly (ADP-ribose) polymerase	C	[^18^F]F-TT	SM	380
	P	[^18^F]F-/[^123^I]iodo-olaparib [^123^I]iodo-MAPi, [^123^I]/ [^124^I]/ [^131^I]iodo-2-PARPi [^18^F]F-PARPi-(FL)	SM SM SM SM	39,381 382 43 383
Glutamate Carboxypeptidase 2	C	[^89^Zr]Zr-IAB_2_M [^18^F]F-DCFPyL [^68^Ga]Ga-PSMA-617 [^68^Ga]Ga-PSMA-11 [^18^F]F-PSMA-1007	Abf Pep Pep Pep Pep	384 385 386 38,82,83,387-389 90
	P	[^18^F]F-DCFPyL [^68^Ga]Ga-PSMA-11	Pep Pep	91 91
Translocator protein (neuronal type) (^$$^)	C	[^11^C]-PK11195 [^18^F]F-GE-180 [^123^I]iodo-CLINDE	SM SM SM	390 38,85,391 392
	P	[^18^F]F-14 (£) [^18^F]F-VUIS1007 [^18^F]DPA-714 [^18^F]F-PBR06 [^18^F]F-VC701 [^18^F]F-AB5186	SM SM SM SM SM SM	393 394 395-499 400,401 402 403
Matrix-metalloproteinases	C	[^131^I]iodo-TM-601	SM	239,404
	P	[^89^Zr]Zr-LEM2/5 [^18^F]F-BR-351 [^18^F]F-P-chlorotoxin [^18^F]F-iCREKA [^68^Ga]Ga-/ [^64^Cu]Cu-MMP-14	Ab SM SM Pep Pep	405 399 406 407 408
Fibroblast activation protein	C	[^68^Ga]Ga-FAPI	SM	63,409,410
	P	[^18^F]F-SiFa_(Glc)_FAPI	SM	411
Lipid metabolism^(++)^	C	[^11^C]-/ [^18^F]F-(ethyl)choline [^11^C]-Acetate	SM SM	30-32,412,413 414,415
	P	[^18^F]-FPIA(*)	SM	416
Fibronectin (neuronal)	C	[^123^I]iodo-L19^(scFv)^_2_	Abf	417
	P	[^18^F]F-iCREKA	Pep	418
Apoptosis	C	[^18^F]F-ML10	SM	419,420
Sigma receptor	C	[^18^F]F-fluspidine (*)	SM	421-423
Somatostatin receptor 2	C	[^68^Ga]Ga-/ [^111^In]In-octreotide [^68^Ga]Ga-octreotide	Pep Pep	58,424 425,426
Deoxycytidine Kinase	C	[^18^F]F-clofarabine	SM	52,427
Neurokinin 1 receptor	C	[^68^Ga]Ga-Substance-P	Pep	93,105
Copper Transporter 1	P	[^64^Cu]Cu-(gold)nanocluster^(+)^	(**)	428
Carbonic Anhydrase IX	P	[^18^F]F-VM4-037	SM	429
Tenascin-C	P	[^99m^Tc]Tc-TTA1 [^18^F]F-/ [^64^Cu]Cu-GBI-10	ON ON	430 431
Histone deacetylases	P	[^18^F]TFAHA 2-[^18^F]BzAHA	SM SM	432 433
Isocitrate Dehydrogenase 1	P	[^18^F]-triazine-diamine [^18^F]F-/ [^131^I]iodo-/ [^125^I]iodo-AGI^5198^ [^18^F]F-/ [^ 125^I]iodo-X (^##^) [^11^C]-Acetate	SM SM SM SM	434 435 436 437
Iron transport	P	[^67^Ga]/ [^68^Ga]Ga-citrate	SM	438
Glutathione transferase	P	[^18^F]F-BuEA-GS	SM	439,440
Hepatocyte growth factor receptor	P	[^89^Zr]Zr-/ [^76^Br]Br-onartuzumab [^89^Zr]Zr-rilotumumab [^64^Cu]Cu-rh-HGF	Ab Ab Pep	441 442 443
Mammalian target of rapamycin	P	[^89^Zr]Zr-transferrin	Prot	44,444
Tyrosine kinases	P	[^18^F]F-dasatinib [^64^Cu]Cu-vandetanib	SM SM	445 445
Myeloid cells	P	[^89^Zr]Zr-anti-CD11b	Ab	446
Platelet-derived growth factor receptor	P	[^68^Ga]Ga-/ [^111^In]In-ZO9591 [^131^I]iodo-/ [^18^F]F-imatinib [^18^F]F-dasatinib	Abf SM SM	369,447 448 445
Stem cells	P	[^64^Cu]Cu-AC133 [^64^Cu]Cu-/ [^89^Zr]Zr-YY146	Ab Ab	449 450,451

($) radiopharmaceutical are grouped as in preclinical (P) and clinical (C) stages of development; chelating agents for radiometal complexation were not denoted in the names to improve clarity of presentation; (++) Fatty acid synthesis (acetate) and choline metabolism for choline; pivalic acid undergoes intracellular metabolism via the fatty acid oxidation pathway (an berrant lipid metabolite detection), (£) no trivial name available- UPAC: 7-chloro-N,N,5-trimethyl-4-oxo-3(6-[^18^F]fluoropyridin-2-yl)-3,5-dihydro-4H-pyridazino[4,5-b]indole-1-acetamide, (^##^) no names given - a small library of nonradioactive analogs were designed and synthesized based on the chemical structure of reported butyl-phenyl sulfonamide enzyme inhibitors, (*) currently in clinical translation, (ç) DNA-based oligonucleotide (aptamer), (°) RNA based oligonucleotide (aptamer), (**) protein-mimic cluster, (+) dual-imaging modality - investigatory (proof-of-concept), (^$$^) expressed on glioma-associated macrophages and microglia, ( ǂ ) vectors: amino-acid (AA), antibody (Ab), antibody fragment (Abf), small biomolecule (SM), peptide (Pep), protein (Prot), oligonucleotide (ON).

**Table 2:** Physical properties and pro/cons of therapeutic radionuclides studied for glioblastoma therapy

Isotope	Range (*in vivo*) (mm)	T ½ (h)	Paired Isotope	Pro's for GB TRT	Cons for GB TRT	Studies in GB	
**^225^Ac** 100.0% ɑ	0.04-0.10	238.10	**^68^Ga**	• *In vivo* range optimal for recurrent/residual GB. • High LET/RBE efficient towards hypoxic GB areals. • DOTA-complexation-simple and universal (some peptides, small molecules and mAb-fragments). • T ½ allows transport; RIT compatible; ideal if no leakage from the target site (upon compound internalization).	• Relatively long T ½ + multiple alpha particles generated (rapid decay chain) → substantial ^225^Ac-based cytotoxicity 105. • Recoiled daughters may influence stability. • Not readily available worldwide.	*C*	**Substance P** (NK-1) 93
						*P*	**E4G10 mAb** (Cadherin 5) 452; IA-TLs (αvβ3 integrin) 453; **Pep-1L** (IL13RA2) 454
**^213^Bi** 2.2% α 97.8% β^-^	0.05-0.10	0.77	**^68^Ga** **^44^Sc**	• *In vivo* range optimal for recurrent/residual GB. • High LET/RBE efficient towards hypoxic GB areals. • DOTA-complexation - simple and universal (some peptides, small molecules and mAb-fragments). • Short T ½ + gamma-energy combination efficient even upon lack of persistent internalization 105. • Availability: ^225^Ac-/^213^Bi-generators. • Energy (440 keV) allows for PK/D assays. • Optimal formulation for intratumoral injection or CED, highly localized radioactive decay *versus* low off target effects 130.	• Short T ½ compromises the residence time required in essential (infiltrating) GB cells, i.e. ratio between cell membrane coverage (receptor affinity) and time is key (Note: irrelevant for intratumoral injection or CED).	*C*	**Substance P** (NK-1) 105,114,241,242
**^211^At** 42.0% ɑ 58.0% EC	0.05 127	7.20 127	^123^I ^76^Br	• *In vivo* range optimal for recurrent/residual GB. • High LET/RBE efficient towards hypoxic GB areals. • Longer T ½ allows for multistep synthetic procedures and transport. • Daughter (^211^Po): emits KX-rays useful for sample counting and *in vivo* scintigraphic imaging 244. • Well-suited for intratumoral injection or CED, highly localized radioactive decay *versus* low off target (systemic) effects 130.	• Limited to mAb (smaller fragments). • Production exclusive to a rare 25-30 MeV cyclotron (± 30 sites worldwide). • Often low biological/chemical stability 455.	*C*	**81C6 mAb G** (tenascin-C) 244
						*P*	**L8A4 mAB** (EGFRvIII) 456
**^131^I** 97.2% β^-^ 2.8% γ	0.80 127	192.00 127	✔	• *In vivo* range (long) efficient on the common GB type (bulky/heterogeneous/2.6-5.0 mm). • Good availability and relatively inexpensive. • Longer T ½ allows transport, compatible for RIT. • Well-understood radiochemistry; universally applicable (peptides, small molecules, mAb). • 10% gamma emission makes it a theranostic (clinical SPECT - or gamma cameras widespread application for patient dosimetry) 260.	• Limited SPECT imaging capacity (suboptimal quantitative imaging); poor spatial resolution (high energy collimators/thick crystal detectors setup). • Radiolabeled proteins degrade rapidly when internalized into tumors; recurrence of [^131^I]iodo-tyrosine and ^131^I-activity in the blood pool → thyroid toxicity plausible.	*C*	**81C6 mAb** (tenascin-C) 98,208,209,446 **BC-2/4 mAb** (tenascin-C) 204,207 **chTNT-1/B mAb** (DNA-histone H1) 236-238 **TM601** 239 **Phenylalanine (IPA)** 458
						*P*	**L19SIP** (Fibronectin) 459,460 **PARPi** (PARP1) 280 **I2-PARPi** (PARP1) 43 **L8A4 mAB** (EGFRvIII) 461,462 **IPQA** (EGFR) 359 **Hyaluronectin glycoprotein** 463 **Phenylalanine** (IPA) 464-466
**^90^Y** 100.0% β^-^	5.30 127	64.10 127	**^68^Ga** **^86^Y** **^111^In**	• *In vivo* range (long) efficient on the common GB type (primary/bulky/heterogeneous/≥ 3 cm). • DOTA-complexation-simple and universal (some peptides, small molecules and mAb-fragments). • Stably retention by GB cells even after endocytosis 108. • Emits highly energetic β-particles 108, ideal for therapy of radioresistant GB. • Longer T ½ allows transport, compatible for RIT.	• Limited efficiency for minimal residual or recurrent GB: needs to be matched with GB tumor size to prevent off target (normal brain) toxicity. • Impractical for nuclear imaging, i.e. high activities (>300 MBq) required (only succeeded for microsphere-based therapies (SIRT) for treating liver tumours 162. • Limited dose administration (preventing nephrotoxic and hematotoxic side effects).	*C*	**Octreotide** (SSTR) 59-61 **Lanreotide** (SSTR) 62 **BC-2/4 mAb** (tenascin-C) 467 **Biotin** 149 **Substance-P** 241
						*P*	**Abegrin** 468
**^177^Lu** 100.0% β^-^	0.62-2.00 127	158.40 127	✔ or **^68^Ga ^89^Zr ^99m^Tc**	• Isotope characteristics capable of affecting GB lesions typically ⌀ < 3 mm diameter 474. • Longer T ½ is compatible with the PK/D and radiochemistry for mAb and proteins 127. • Fairly straightforward conjugation chemistry 127,470. • Good availability and low cost 469. • Emission of low-energy gamma - true theranostic 127. • [^177^Lu]Lu-mAb: higher specificity index (i.e. less non-specific cell killing) than analogous [^90^Y]Y-mAb 156.	• Moderately nephrotoxic and hematotoxic (< ^90^Y).	*C*	**Substance-P** (NK-1) 241 **PSMA-617** 84,86
						*P*	**6A10 Fab** (CAXII) 471 **CXCR4-L** (CXCR4) 472 **VH-DO33** (LDLR) 473 **2.5D/2.5F** (Integrin) 474 **L8A4 mAb** (EGFRvIII) 475,476 **IIIA4 mAb** (EphA3) 77
**^188^Re** 100.0% β^-^	5.00-10.8	16.98	✔	• *In vivo* range (long) efficient on the common GB type (primary/bulky/heterogeneous/≥ 3 cm). • Readily available and inexpensive via ^188^W-/^188^Re-generator (carrier-free, high specific activity). • Gamma emission suitable for imaging (better image quality than^ 186^Re).	Unfavorably-low energy characteristics 114. Radioactive source material for generator production: Reactor-based ^188^W production only in 2-3 reactors worldwide 482.	*C*	**Nimotuzumab** (EGFR) 248,483
						*P*	**PEG-nanoliposome** 440 **BMSC implantation** 479 **Nanocarriers** (CXCR4) 150 **Lipid nanocapsules** 480,481 **Microspheres in fibrin glue gel** 482 **U2 DNA aptamer** (EGFRvIII) 483,484
**^64^Cu** 18.0% β^+^ 39.0% β^-^ 42.5% **EC** 0.5% γ	β 1.00 AE 0.13 485	12.70	✔	• Readily available. • Radiometal complexation well understood and universally applicable (most peptides/mAb/small molecules and nanoparticles). • Combined β^+^/β^-^ emission makes it a true theranostic. • Radioisotope salts ([^64^Cu]CuCl_2_): the higher intratumoral accumulation of Cu correlates with overexpression of human copper transporter 1 (hCTR1) in GB cancer cells 486. • AE cascade from EC are considered high LET radiation with ~ 2 keV of average energy 485.	• Radiometal complexation can be unstable *in vivo* 486,487. • Lack of radiometal-specific chelating agents. • Radiation dosimetry: complex decay scheme affects absorbed dose from high-LET AE emissions 485.	*P*	**CuCl_2_** 54,75,184,498,489 **Cyclam-RAFT-c(RGDfK)4** (αvβ3 integrin) 54 **Pep-1L** (IL13RA2) 454 **ATSM** (Hypoxia) 75 **IIIA4 mAb** (EphA3) 77 **TNYL-RAW** (EPHR) 40 **1C1 mAb** (EphA2) 362
**^67^Cu** 100.0% β^-^	0.20	62.40	✔ or **^64^Cu**	• Treats small residual or recurrent GB lesions (⌀ ≤5 mm) 56. • Combined β^+^/β^-^ emission makes it a true theranostic. • Supports SPECT imaging of patient dosimetry 490. • Biochemistry of copper is well studied; radiometal complexation well understood and universally applicable (most peptides/mAb/small molecules and nanoparticles) 56,491. • No off-target toxicity reported (bone or organs). • Radioisotope salts ([^67^Cu]CuCl_2_): the higher intratumoral accumulation of copper correlates with overexpression of human copper transporter 1 (hCTR1) in GB cancer cells 486.	• Large amounts rarely available; limits research and clinical trial design 491.	*P*	**RAFT-c(RGDfK)4** (αvβ3 integrin) 56
**^125^I** 100.0% EC	0.002	1425.60	**^111^In**	• Isotope applicable in brachytherapy for GB. • Systemic immune-therapy well tolerated 163.	• Very long T½ may impose limitations for clinical use (radioprotection, therapeutic efficacy, slow dose rate). • Gamma emission energy not siutable for nuclear imaging. • Range and energy is not effective for heterogeneous radioresistent GB.	*C*	**425 mAb** (EGFR) 163,166,167,227,492,495
						*P*	**L8A4 mAB** (EGFRvIII) 499,500 **UdR** 165,496 **806 mAb** (EGFRvIII) 363
**^123^I** 97.0% EC3.0% γ	0.001-0.01	13.20	✔	• Short T ½ and gamma emission energy suitable for scintigraphic imaging *in vivo.* • More suitable choice for potential use in RIT (as to ^125^I) 156.	• Not widely available (<^131^I). • T ½ is not compatible for PK/D investigation.	*P*	**MAPi** (PARP1) 382
**^111^In** 100.0% EC	0.04	67.20	✔	• Characteristic suitable for *in vitro* GB studies. • True theranostic: gamma emission energy allows scintigraphic imaging *in vivo*.	• Complexation chemistry required; incorporation kinetics slow for radiolabeling mAb (no direct radiometal conjugation).	*P*	**GA17 Ab** (α3 integrin) 497 **806 mAb** (EGFRvIII) 497

(✔) Theranostic radionuclide, (*) human case study, convection enhanced delivery (CED), pharmacokinetic/dosimetry studies (PK/D), glioblastoma (GB), radioimmunotherapy (RIT), oxygen enhancement ratio (OER), polyethylene glycol (PEG), Bone-marrow mesenchymal stem cells (BMSC)**,** electron capture (EC), linear energy transfer (LET), Auger electron (AE), single-photon emission computed tomography (SPECT), physiological half-life (T ½ ).

**Table 3:** Overview and characteristics of different rodent tumor models for glioblastoma imaging

Model	Methodology	Pro	Con	Cell lines/models	References
ENU-induced	• Exposure *in utero* to ENU (DNA damage induces brain tumors embryos); • Dissection and culturing of these tumors *in vitro* to create animal GB models.	• Immunotherapeutic research tool. • Commercially available. • Extensively studied. • Provides genetic brain heterogeneity, micro-environment • Intact immune system and BBB.	• Often ENU tumor characteristic differs from human GB; • GB tumor formation poorly reproducible.	C6, 9L, T9, RG2, F98, BT4C, and RT-2	278,283,498-504
GEMM	• Gene mutations result in spontaneous tumor formation; • Transgenic mouse lines are commonly derived by direct pronuclear microinjection of transgenes into fertilized oocytes, followed by implantation into pseudo-pregnant females; • Gene targeting of embryonic stem cells by electroporation; • Viral-mediated methods; • Cre recombinase transgenics	• Close genetic resemblance to human GB tumors: suitable to investigate behavior of genetically defined gliomas. • Identify the molecular events responsible for tumor initiation and progression. • Analyze the role of the microenvironment • Studies on drug distribution to glioma cells in the brain.	• Does not completely reflect the intratumoral genomic and phenotypic heterogeneity; • Tumor initiation cannot be controlled.	EGFR amplification/Ras-gene activation (classical GB); NF1 depletion (mesenchymal GB); PDGF amplification (proneural GB)	276,278,279,284,504-506
PDX	• Surgically obtained human glioma specimens. After preparing cell/tissue cultures these can also be implanted heterotopically or orthotopically in immunocompromised rodents; • Immediate implantation of surgically obtained material into the brain of the animal	• Recapitulate genetic and phenotypic features of the original tumor	• Relatively low engraftment and variable growth rate hamper standardization and experimental planning. • Requires immunodeficient animals.	IDH1^R132H^-E478	276,285,504
PDGC		• High engraftment and growth rates; • Good reproducibility; • Reliable disease growth and progression	• Does not recapitulate genetic and phenotypical features of original tumor. • Requires immunodeficient animals	U87, and U251	

Footnotes and abbreviated content: Ethyl-nitrosourea (ENU)-induced gliomas, genetically engineered models (GEMM) and patient-derived xenograft or glioma cell models (PDX or PDGC), platelet-derived growth factor (PDGF), blood brain barrier (BBB), glioblastoma (GB), neurofibromatosis type 1 gene (NF1), epidermal growth factor receptor (EGFR), deoxyribonucleic acid (DNA).

## Abbreviations

AE: Auger electrons
AMT: adsorptive-mediated transcytosis
BBB: blood-brain barrier
CED: convection enhanced delivery
CMT: carrier-mediated transport
CNS: central nervous system
CPP: cell-penetrating peptides
CXCR4: Chemokine receptor 4
DSB: double strand breaks
DTPA: diethylenetriamine pentaacetate
EGFR: epidermal growth factor receptor
EBRT: external beam radiotherapy
ENU: ethyl-nitrosourea
EPHR: Ephrin receptors
FAPI: fibroblast activation protein
FDA: Food and Drug Administration
GB: glioblastoma
GEMMs: genetically engineered models
GMP: Good Manufacturing Practice
IDH: isocitrate dehydrogenase
IMPD: investigational medicinal product dossier
ITLC: instant thin layer chromatography
LET: linear energy transfer
MA: molar activity
mTOR: mammalian target of rapamycin
MIRD: medical internal radiation dose
MMP: Matrix-metalloproteinases
MRI: magnetic resonance imaging
PARP: poly (ADP-ribose) polymerase
PDX/PDGC: patient-derived xenograft or glioma cell models
PET: positron emission tomography
PRRT: peptide receptor radionuclide therapy
PSMA: prostate-specific membrane antigen
PTEN: phosphatase and tensin homolog
RBE: relative biological effectiveness
RGD: Arginine-glycine-aspartate
RIBE: radiation-induced bystander effects
RIT: radio-immunotherapy
RMT: receptor-mediated transport
RN: radiation necrosis
RT: radiation therapy
SA: specific activity
SCRC: surgically created resection cavity
SP: substance-P
SPECT: single photon emission tomography
SSR2: somatostatin receptor 2
TERT: telomerase reverse transcriptase
TME: tumour micro-environment
TMZ: temozolomide
TRT: targeted radionuclide therapy
TSPO: translocator protein
VEGFR: vascular endothelial growth factor receptor
WHO: world health organization
